# Identifying distinct service use phenotypes across levels of suicidality and self-harm: a UK Biobank study

**DOI:** 10.1038/s44184-025-00160-8

**Published:** 2025-09-23

**Authors:** Biya Tang, Fiona Shand, Helen Christensen, Alexis E. Whitton

**Affiliations:** Black Dog Institute, University of New South Wales, Sydney, NSW Australia

**Keywords:** Psychology, Health services

## Abstract

Mental health service use among individuals experiencing suicidality and self-harm is highly variable, with many individuals not seeking professional help. Using large-scale data from the UK Biobank, this study examined factors predictive of distinct service use phenotypes across varying levels of suicidality and self-harm. Male sex, the absence of psychological distress, and lower severity of suicide risk were the factors most strongly associated with reduced engagement with professional care. Additionally, although use of informal support predicted greater odds of service engagement, the type of support varied depending on suicidality severity: informal therapeutic strategies (e.g. yoga) were linked to service use among lower-risk individuals, whereas use of helplines was most strongly associated with service use among higher-risk individuals (e.g. those with a history of suicide attempts). These findings suggest that tailoring interventions to the unique needs and contexts of different subgroups may improve service engagement among individuals experiencing suicidality and/or self-harm.

## Introduction

Suicidal and self-harm behaviours are major global health crises, leading to substantial social and financial impacts on individuals, their families and broader communities^1–3^. Increasing engagement with services that provide evidence-based mental health interventions, including psychological, behavioural and/or pharmacological therapies^4–6^, is crucial for managing and preventing suicidal and self-harm behaviours. Despite the increasing accessibility of mental health care^7,8^, converging evidence indicates that a significant proportion of individuals experiencing suicidal thoughts and/or self-harm either do not seek treatment or do not perceive a need for professional help^9–14^. For example, over two-thirds of individuals who died by suicide had no contact with mental health services in the 12 months prior to death^13^. Identifying the characteristics of those who do not seek help is essential for improving strategies to connect at-risk individuals with timely support.

A growing body of research has sought to identify factors characterising subgroups of suicidal individuals who are less likely to use professional services^11,14–23^. Prior studies have consistently shown that males^14,16,17,19^, those without a mental health disorder diagnosis, those with less severe suicidality, and those with lower levels of psychological distress^15,16,18,19,21–23^, are less likely to use mental health services. Other studies have also shown that individuals who are employed^24^ and those with better physical health^14^, are less likely to engage with treatment for suicidality. Additionally, studies show that those who do not readily use informal support (e.g. support from family/friends) may also be less likely to use formal mental health services^11,15,16,23^. Together, these findings suggest that efforts aimed at enhancing service use among those with suicidality may need to be tailored towards the needs of specific subgroups of individuals.

Despite valuable insights from prior studies, critical gaps remain. First, existing literature rarely distinguishes between treatment seeking and treatment receipt, often categorising the ‘use of mental health services’ as a single entity^10,11,17,20–23^. Although inherently linked, treatment seeking and treatment receipt are distinct stages of the service contact process. Factors predictive of treatment receipt may not necessarily predict treatment seeking, and vice versa. For example, suicidal individuals may receive treatment without actively seeking it, such as in cases of involuntary hospitalisation after a nonfatal suicide attempt^25^. Conversely, after initial contact with a mental health service, various factors (e.g. cost, time, preferences, service characteristics) may lead individuals to forgo receiving the treatment initially sought. A more comprehensive understanding of these factors is essential to design suicide prevention efforts that effectively promote both treatment seeking and subsequent treatment receipt.

Another important gap in the literature is understanding how heterogeneity in suicidality impacts service contact. Ideation-to-action theories such as the interpersonal theory of suicide and the three-step theory emphasise that suicide ideation and suicidal behaviour are distinct processes with different risk factors and mechanisms^26^, highlighting the importance of stratifying individuals by suicidality subgroups. Yet, existing research has predominantly focused on individuals at a single level of suicide risk, limiting comparison of predictors of service contact across groups with different degrees of suicidality^10,14,15,18–21,23^. The few studies that have focused on predictors of service contact in different subgroups of suicidal individuals consist largely of reviews^11,16,21,22^, or cross-sectional studies that either focus on a few selected predictors^15,19^ or use small sample sizes^17,18,20,23^. Large-scale datasets are needed to extend this work and provide a comprehensive assessment of the diverse factors predictive of service use among different subgroups of suicidal individuals. Furthermore, longitudinal data is needed to confirm the temporal direction of the relationship between predictive factors and subsequent service contact.

The current study aimed to address these research gaps by using large-scale data from the UK Biobank (UKB) to examine associations between a comprehensive range of sociodemographic, health-related, and help-related factors, and different treatment-related behaviours, across the spectrum of suicidality. In this study, we use the term 'service use phenotypes' to refer to subgroups of individuals who share similar treatment-related behaviours. The application of the term is consistent with suicide prevention literature focusing on subgroups with similar behavioural characteristics^27–29^. We examined whether similar or distinct factors were predictive of treatment seeking and treatment receipt. In addition, we examined whether factors predictive of these treatment-related behaviours overlapped or differed among subgroups of individuals with different forms of suicide and self-harm behaviours. Given that various factors have been linked to lower rates of service use^14–23^, we hypothesised that there would be distinct subgroups of at-risk individuals who are less likely to engage with mental health services. Capitalising on the longitudinal structure of the UKB data, we complemented our primary cross-sectional analysis with exploratory longitudinal analyses to assess factors predictive of changes in future treatment seeking and receipt.

## Methods

### Participants

The UKB (http://www.ukbiobank.ac.ak) is a prospective study with a large general population cohort of approximately 502,000 adults aged 40–69 years. Baseline assessments occurred between 2006 and 2010, covering sociodemographic characteristics, lifestyle, mental and physical health. After baseline assessments, a series of online questionnaires were regularly released for continuous collection of comprehensive data on health outcomes, including the Mental Health Questionnaire (MHQ) in 2016, Digestive Health Questionnaire (DHQ) in 2018 and Mental Wellbeing Questionnaire (MWQ) in 2022. UKB data contain linked health data through multiple national registries, including hospital inpatient data and mortality data (for mortalities occurring in the UK). Informed consent for the assessments and the register linkage was obtained for all participants. Participants were included in the analysis if they displayed suicidality and/or self-harm behaviours as indicated by the questionnaires (see below), inpatient hospital data, or mortality records, and responded to at least one of the questions assessing treatment-related behaviours. Thus, the final sample for analysis consisted of 15,966 participants.

Ethics approval for the UKB project was granted by the NHS National Research Ethics Service (16/NW/0274). The current study received approval from the UK Biobank (application #92794), and the study protocol was pre-registered on Open Science Framework (osf.io/2upbh).

### Measures

The UKB items used for assessing each variable, including the corresponding field codes, and item sources, can be found in Supplementary Table 1.

Suicidal ideation (SI) was assessed in 2016 by the MHQ question 'Over the last 2 weeks, how often have you been bothered by any of the following problems? Thoughts that you would be better off dead or of hurting yourself in some way?' To determine if an individual had ever had a suicide attempt (SA), they were asked, 'Have you harmed yourself with the intention to end your life?'.

Death by suicide at any time during the study period was defined as 'all deaths from intentional self-harm for persons aged 10 years and over, and deaths caused by injury or poisoning where the intent was undetermined for those aged 15 years and over' (UK Office for National Statistics). This was ascertained via linkage with the mortality data using ICD-10 codes X60-84 (intentional self-harm) and Y10-Y34 (undetermined intent), excluding Y33.0 (unspecified place). To enhance the accuracy in identifying deaths resulting from intentional self-harm rather than an accident, cases coded under Y10-Y34 (undetermined intent) without a report of SI or SA in the MHQ were excluded from the death by suicide subgroup (67 participants were excluded during this process).

Self-harm was partly ascertained in 2016 by the MHQ question 'Have you deliberately harmed yourself, whether or not you meant to end your life?' In addition to self-reported data, self-harm cases were also identified through hospital admissions at any time during the study period with a diagnosis of intentional self-harm (ICD-10 ×60-X84, ICD-9 E950-958) and injury/poisoning of undetermined intent (ICD-10 Y10-34 excluding Y33.9, ICD-9 980-989 excluding 988.8). To ensure that the ‘self-harm’ behaviour coded under Y10-Y34 or E980-E989 (undetermined intent) was deliberate, cases without self-reported self-harm in the MHQ were excluded from the self-harm subgroup.

Based on participants’ responses to the MHQ, hospital records and the mortality records, four subgroups were derived: (1) SI only—participants with SI but no prior SA, who are alive or, if deceased, did not die as a result of suicide; (2) SI with SA—participants with SI and prior SA, who are alive or, if deceased, did not die as a result of suicide; (3) Death by suicide; and (4) Self-harm.

Three treatment-related behaviours were derived from the MHQ questions in 2016. Treatment seeking (yes/no) was determined by participants’ responses to two questions asking whether they had ever disclosed their symptoms of depression/anxiety to a professional. Treatment receipt (yes/no) was determined by responses to four MHQ items assessing the use of activities and substances in response to depression and/or anxiety symptoms. Those who indicated having attended formal therapies such as psychotherapy, and used prescribed medication, were categorised as having received treatment.

Other treatment-related behaviours (yes/no) were assessed in 2016 by the MHQ question 'Have you sought or received professional help for mental distress?' and two similar items in the DHQ 'Have you ever been offered/sought treatment for depression/anxiety?'. Since the DHQ was administered two years after the MHQ, those items were used as a replication of the assessment of other treatment-related behaviours originally assessed by the MHQ. If a participant did not respond to any of the above treatment-related measures, the participant was excluded from the analysis.

We selected the following variables as potential predictors of treatment-related behaviours among individuals experiencing suicidality and/or self-harm because they are well-established correlates of help-seeking and engagement in mental health services in prior research. Potential explanatory variables included help-related, sociodemographic, and health-related factors. Detailed information about the categorisation and recoding of these variables are provided in the Supplementary Materials.

Help-related variables were collected through the MHQ in 2016, and include receipt of informal therapeutic strategies (e.g. yoga and art classes), OTC medication, self-medication with alcohol/drugs, use of helpline, and use of interpersonal support (e.g. family/friends). Notably, use of helpline and interpersonal support were assessed specifically regarding actions following self-harm in the MHQ, and thus were considered as potential predictors for the subgroups with self-harm behaviours, i.e. the SI with SA subgroup and the Self-harm subgroup.

Sociodemographic variables were all assessed at baseline, including sex (male/female), age at recruitment, ethnicity, educational attainment, employment status, household income, neighbourhood deprivation, living arrangement, location and confiding.

Health-related variables include perceived overall health (measured at baseline), mental health diagnosis (yes/no), psychological distress (yes/no), addiction (yes/no) and number of self-harm methods used (reported in 2016).

A symptom severity score was calculated and served as a covariate in the analyses. This composite depression/anxiety score was derived by summing the total scores of the PHQ-9 (excluding the item assessing suicidal ideation) and GAD-7 administered in 2016, ranging from 0 to 45. These scores were then standardised (mean = 0, SD = 1), with higher scores indicating more severe depression/anxiety symptoms.

### Statistical analysis

This section outlines the primary approaches used in the main and exploratory analyses. Detailed descriptions of analyses, data handling procedures, and tests for multicollinearity are provided in the Supplementary Materials.

For the planned analyses, cross-sectional multivariable logistic regression models were performed to investigate associations between the explanatory factors and treatment-related behaviours (treatment seeking, treatment receipt, and other treatment-related behaviours) in the total sample first, and then in each subgroup. The effect size of each explanatory variable was compared across subgroups using two-sample z-tests. Symptom severity was included as a covariate in all planned analyses. Given the widely reported sex differences in treatment-related behaviours, e.g. refs. ^16,18^, we also conducted exploratory analyses stratifying by sex. To control for multi-testing, Principal Component Analysis was performed on the correlation matrix of common explanatory variables across all groups to determine the number of principal components explaining 99.5% of the variance (*n* = 25). Then a Bonferroni-adjusted *p* value was calculated as. 05/25. As such, results were considered statistically significant at *P* < 0.002. All analyses were conducted in R version 4.4.1.

Due to unexpected findings from the planned analyses, the following exploratory analyses were conducted to provide a more accurate and comprehensive examination of factors that were differentially associated with treatment-related behaviours across suicidality subgroups. Details of these analyses are provided in the Supplementary Materials. First, as no model could be run for the Death by Suicide subgroup in the planned analyses due to low completion rates of the MHQ, we further explored factors associated with baseline treatment seeking in the Death by Suicide subgroup, and other subgroups. Second, sensitivity analyses removing the absence/presence of a mental health disorder were conducted as part of the primary analyses. For the Self-harm subgroup, this involved two nested models, with self-harm-specific variables excluded in the base model and added in the full model. Third, analyses assessing change in suicidality severity and treatment behaviours over time were conducted and focused on changes in suicidality severity, as well as changes in treatment seeking and receipt from 2016 to 2022. Fourth, we differentiated non-suicidal self-injury (NSSI) and suicidal self-injury (SSI) based on responses to the MHQ questions assessing SI and SA, and examined associations between treatment-related behaviours and explanatory factors in those subgroups. Additional analyses were performed on self-harm methods, including comparing the usage of each method across different levels of treatment-related behaviours.

## Results

### Sample characteristics

We used data from the subset of *N* = 15,966 individuals in the UKB who reported experiencing SI, a prior SA, or a prior hospitalisation due to intentional self-harm, as well as those who had died by suicide. Sample characteristics are reported in Supplementary Table 2. The sample was predominantly White British (*n* = 13,596, 85.5%), over half were female (*n* = 9597, 60.1%), and were on average, 53 years old (M = 53.14; SD = 7.85). The majority (*n* = 8247, 67.3%) had a mental health disorder diagnosis. A substantial majority indicated having sought (*n* = 9103, 80.9%) and/or received (*n* = 8554, 75.8%) treatment for mental health problems. Among the suicidality subgroups—SI only, SI with SA, and death by suicide—the proportion of those with a mental health diagnosis ranged from 62% (SI Only) to 89% (SI with SA), and the proportion of those experiencing psychological distress ranged from 67% (SI Only) to 94% (SI with SA). Treatment-related behaviours also differed between subgroups, with all types of treatment-related behaviours being more common in the SI with SA subgroup than in the SI Only subgroup.

### Factors associated with different stages of service contact

We conducted preliminary analyses to evaluate the nature and extent of the available data relevant to our pre-registered analysis (see Methods), which aimed to identify factors predictive of treatment seeking and treatment receipt among at-risk individuals. Preliminary results (see Supplementary Materials) indicated significant overlap between one of these predictive factors (whether an individual had a mental health disorder diagnosis) and the treatment-related variables (treatment seeking: χ^2^ = 3517.86, φ_c_ = .60, *P* < 0.001; treatment receipt: χ^2^ = 3847.37, φ_c_ = .62, *P* < 0.001; Supplementary Table 3). To avoid masking the effects of other predictors, we conducted sensitivity analyses excluding this factor. Furthermore, preliminary analysis indicated the need for a nested modelling approach for sensitivity analyses in the Self-harm subgroup, as self-harm-specific variables were only assessed among self-harming individuals. As such, in the base model, all relevant explanatory variables were included, except for self-harm-specific variables and the absence of a mental health diagnosis. In the full model, we added the self-harm-specific variables for the self-harm subgroup.

**Fig. 1:**
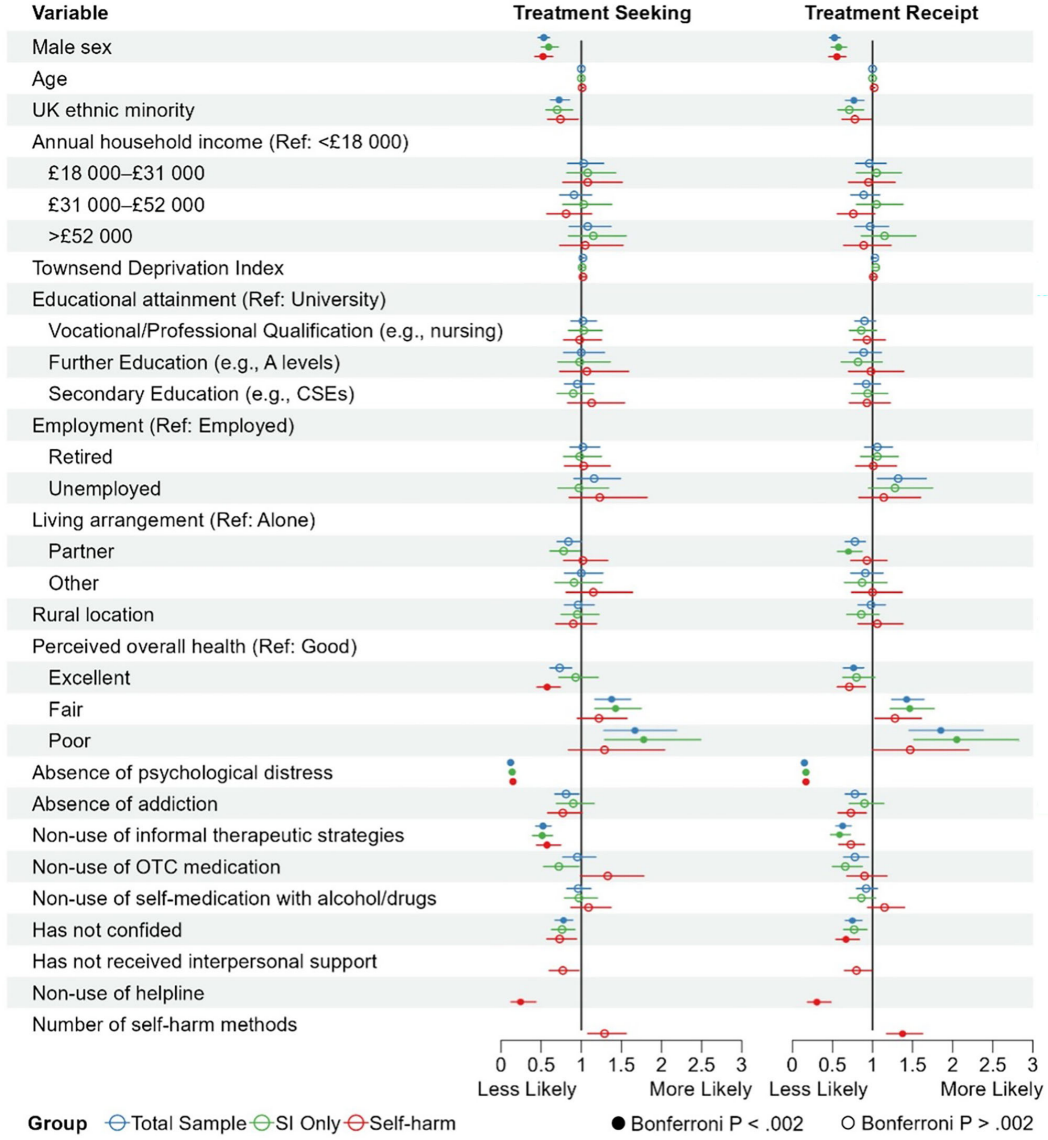
Factors associated with treatment seeking and receipt across subgroups. Forest plots showing the odds ratios (with 95% confidence intervals) for each factor in the primary multivariable models, examining their associations with treatment seeking and treatment receipt, respectively, across the total sample, suicidal ideation only (SI Only) and self-harm subgroups. Odds ratios and 95% confidence intervals for the self-harm subgroup were derived from the full model, which included self-harm-specific variables. Figure symbols: Blue circles = Total sample; green circles = SI Only subgroup; red circles = Self-harm subgroup; filled in circles = significant at Bonferroni-corrected threshold (*P* < 0.002); empty circles = not significant at Bonferroni-corrected threshold (i.e. *P* > 0.002).

Forest plots displaying odds ratios and 95% confidence intervals for the associations between each factor and treatment seeking and receipt derived from sensitivity models are shown in Fig. 1.

### Treatment seeking

Effect sizes (odds ratios) for each factor predicting treatment seeking are reported in Table 1. Across the entire sample, the following factors were linked to reduced odds of treatment seeking: being male (vs. female), absence (vs. presence) of psychological distress, non-use (vs. use) of informal therapeutic strategies (e.g. yoga and art classes), minority ethnicity (vs. White British), absence (vs. presence) of confiding, good perceived overall health (vs. fair and vs. poor) and excellent perceived overall health (vs. good).

**Table 1:** Effect sizes and 95% confidence intervals for each explanatory variable, excluding absence of a mental health diagnosis analysed in the multivariable logistic regression model assessing associations with treatment seeking for the total sample, the SI only subgroup, and the self-harm subgroup

Outcome: treatment seeking	Total sample (*n* = 15,966)		SI only (*n* = 5809)		Self-harm (*n* = 10,442)			
					Model 1 (Base model)		Model 2 (Full model)	
Variable	**Odds ratio**	**95% CI**	**Odds ratio**	**95% CI**	**Odds ratio**	**95% CI**	**Odds ratio**	**95% CI**
Sex (Ref = Female)								
Male	**0.53**	**[0.46,0.60]**	**0.59**	**[0.50, 0.71]**	**0.53**	**[0.43, 0.65]**	**.52**	**[0.42, 0.64]**
Age (years)	1.00	[0.99, 1.01]	1.00	[0.99, 1.02]	1.01	[1.00, 1.03]	1.01	[1.00,1.03]
Ethnicity (Ref = White British)								
Ethnic Minority	**0.73**	**[0.62, 0.87]**	0.70	[0.56, 0.89]	.73	[0.57, 0.94]	0.74	[0.58, 0.96]
Annual Household Income (Ref = <£18 000)								
£18 000–£31 000	1.03	[0.83, 1.28]	1.08	[0.82, 1.43]	1.11	[0.80, 1.55]	1.08	[0.77, 1.51]
£31 000–£52 000	0.91	[0.73, 1.13]	1.03	[0.77, 1.38]	0.83	[0.60, 1.16]	0.81	[0.57, 1.13]
>£52 000	1.08	[0.85, 1.37]	1.15	[0.84, 1.56]	1.09	[0.76, 1.60]	1.05	[0.73, 1.52]
Townsend deprivation index	1.02	[1.00, 1.04]	1.01	[0.98, 1.04]	1.02	[0.99, 1.05]	1.02	[0.99, 1.05]
Educational attainment (Ref = University)								
Vocational	1.02	[0.87, 1.19]	1.03	[0.84, 1.26]	1.04	[0.83, 1.32]	.98	[0.78, 1.25]
Further	1.00	[0.78, 1.29]	0.98	[0.71, 1.36]	1.04	[0.72, 1.55]	1.07	[0.73, 1.59]
Secondary	0.95	[0.79, 1.16]	0.90	[0.70, 1.15]	1.19	[0.88, 1.62]	1.13	[0.83, 1.54]
Employment (Ref = Employed)								
Retired	1.02	[0.86, 1.23]	0.98	[0.78, 1.25]	1.06	[0.81, 1.40]	1.03	[0.79, 1.36]
Unemployed	1.16	[0.91, 1.49]	0.97	[0.71, 1.34]	1.37	[0.95, 2.02]	1.23	[0.85, 1.82]
Living Arrangement (Ref = Alone)								
Partner	0.84	[0.70, 1.00]	0.78	[0.61, 0.98]	0.97	[0.74, 1.2]	1.02	[0.78, 1.33]
Other	1.00	[0.79, 1.27]	0.91	[0.67, 1.26]	1.15	[0.81, 1.63]	1.15	[0.81, 1.64]
Location (Ref = Urban)								
Rural	0.96	[0.79, 1.16]	0.95	[0.75, 1.22]	0.87	[0.66, 1.15]	0.90	[0.68, 1.19]
Confiding (Ref = Presence)								
Absence	**0.77**	**[0.66, 0.89]**	0.76	[0.63, 0.92]	0.74	[0.59, 0.94]	0.74	[0.58, 0.94]
Perceived overall health (Ref = Good)								
Excellent	**0.73**	**[0.61, 0.88]**	0.93	[0.72, 1.21]	**0.60**	**[0.47, 0.78]**	**0.58**	**[0.45, 0.75]**
Fair	**1.38**	**[1.18, 1.62]**	**1.43**	**[1.17, 1.75]**	1.26	[0.99, 1.63]	1.22	[0.95, 1.57]
Poor	**1.67**	**[1.28, 2.19]**	**1.78**	**[1.29, 2.49]**	1.36	[0.89, 2.13]	1.29	[0.84, 2.04]
Psychological distress (Ref = Presence)								
Absence	**0.13**	**[0.12, 0.15]**	**0.14**	**[0.12, 0.17]**	**0.15**	**[0.12, 0.17]**	**0.15**	**[0.12, 0.18]**
Addiction (Ref = Presence)								
Absence	0.81	[0.67, 0.97]	0.90	[0.69, 1.16]	0.73	[0.55, 0.95]	0.76	[0.58, 1.01]
Informal therapeutic strategies (e.g. yoga; Ref = Use)								
Non-use	**0.53**	**[0.44, 0.63]**	**0.52**	**[0.40, 0.65]**	**0.54**	**[0.42, 0.70]**	**0.58**	**[0.45, 0.75]**
OTC medication (Ref = Use)								
Non-use	0.96	[0.77, 1.18]	0.72	[0.53, 0.97]	1.30	[0.97, 1.73]	1.33	[0.99, 1.78]
Self-medication with alcohol/drugs (Ref = Use)								
Non-use	0.96	[0.82, 1.12]	0.97	[0.79, 1.20]	1.01	[0.80, 1.26]	1.09	[0.87, 1.37]
Non-clinical care (e.g. helplines; Ref = Use)								
Non-use		N/A		N/A		N/A	**0.24**	**[0.12, 0.43]**
Interpersonal support (e.g. family; Ref = Use)								
Non-use		N/A		N/A		N/A	0.77	[0.61, 0.97]
Number of self-harm methods used (0–6)		N/A		N/A		N/A	1.29	[1.08, 1.56]

In the total sample and the SI only subgroup, self-harm-related variables (i.e. non-clinical care for self-harm, interpersonal support for self-harm, and number of self-harm methods used) were not included in the multivariable regression model as a large proportion of participants did not self-harm. Regression models could not be run for the SI with SA and Death by Suicide subgroups due to a heavily imbalanced ratio of treatment seekers vs. non-seekers (SI with SA) and insufficient data (death by suicide). Bold values denote significance based on a Bonferroni-corrected *P* threshold of 0.002.

When examining these factors within specific suicidality subgroups:

In the SI Only subgroup, male sex, absence of psychological distress, non-use of informal therapeutic strategies, and good perceived overall health (vs. fair and vs. poor), were associated with reduced odds of treatment seeking.

In the self-harm subgroup, the base model revealed that male sex, absence of psychological distress, non-use of informal therapeutic strategies, and excellent perceived overall health (vs. good), were associated with reduced odds of treatment seeking. In the full model where self-harm-specific variables were added, non-use of helplines (vs. use) was an additional factor linked to reduced odds of treatment seeking.

### Treatment receipt

Effect sizes (odds ratios) for each factor predicting treatment receipt are reported in Table 2. Across the entire sample, similar factors were associated with reduced odds of treatment receipt as were associated with reduced odds of treatment seeking, namely, male sex, absence of psychological distress, non-use of informal therapeutic strategies, minority ethnicity, absence of confiding, good perceived overall health, and excellent perceived overall health.

**Table 2:** Effect sizes and 95% confidence intervals for each explanatory variable, excluding absence of a mental health diagnosis analysed in the multivariable logistic regression model assessing associations with treatment receipt for the total sample, the SI only subgroup, and the self-harm subgroup

Outcome: Treatment receipt	Total sample (*n* = 15,966)		SI only (*n* = 5809)		Self-harm (*n* = 10,442)			
					Model 1 (Base model)		Model 2 (Full model)	
Variable	**Odds ratio**	**95% CI**	**Odds ratio**	**95% CI**	**Odds ratio**	**95% CI**	**Odds ratio**	**95% CI**
Sex (Ref = Female)								
Male	**0.52**	**[0.46, 0.59]**	**0.57**	**[0.48, 0.67]**	**.56**	**[0.46, 0.67]**	**0.55**	**[0.45, 0.66]**
Age (years)	1.00	[0.99, 1.01]	1.00	[0.99, 1.01]	1.01	[1.00, 1.03]	1.02	[1.00, 1.03]
Ethnicity (Ref = White British)								
Ethnic minority	**0.77**	**[0.66, 0.90]**	0.71	[0.57, 0.89]	0.77	[0.62, 0.97]	0.78	[0.62, 0.98]
Annual household income (Ref = <£18 000)								
£18 000–£31 000	0.96	[0.79, 1.17]	1.05	[0.80, 1.36]	0.97	[0.72, 1.30]	0.95	[0.70, 1.28]
£31 000–£52 000	0.89	[0.73, 1.09]	1.05	[0.80, 1.38]	0.78	[0.57, 1.04]	0.76	[0.56, 1.03]
>£52 000	0.97	[0.78, 1.20]	1.15	[0.86, 1.54]	1.09	[0.76, 1.56]	0.89	[0.64, 1.23]
Townsend deprivation index	1.03	[1.01, 1.05]	1.04	[1.01, 1.07]	0.91	[0.66, 1.26]	1.01	[0.98, 1.04]
Educational attainment (Ref = University)								
Vocational	0.90	[0.78, 1.04]	0.86	[0.71, 1.05]	0.97	[0.79, 1.20]	0.93	[0.76, 1.16]
Further	0.89	[0.71, 1.11]	0.82	[0.61, 1.12]	0.97	[0.70, 1.37]	0.98	[0.70, 1.39]
Secondary	0.92	[0.77, 1.10]	0.94	[0.74, 1.19]	0.97	[0.75, 1.26]	0.93	[0.71, 1.22]
Employment (Ref = Employed)								
Retired	1.06	[0.90, 1.25]	1.06	[0.85, 1.32]	1.04	[0.81, 1.32]	1.01	[0.79, 1.30]
Unemployed	1.32	[1.06, 1.67]	1.28	[0.95, 1.75]	1.24	[0.91, 1.73]	1.15	[0.83, 1.60]
Living arrangement (Ref = Alone)								
Partner	0.78	[0.66, 0.91]	**0.69**	**[0.55, 0.86]**	0.90	[0.71, 1.15]	0.93	[0.73, 1.18]
Other	0.91	[0.73, 1.13]	0.87	[0.65, 1.18]	1.02	[0.75, 1.40]	1.00	[0.74, 1.37]
Location (Ref = Urban)								
Rural	0.98	[0.82, 1.16]	0.86	[0.68, 1.08]	1.04	[0.81, 1.35]	1.06	[0.82, 1.38]
Confiding (Ref = Presence)								
Absence	**0.76**	**[0.66, 0.87]**	0.77	[0.64, 0.93]	**0.67**	**[0.54, 0.84]**	**0.67**	**[0.54, 0.84]**
Perceived overall health (Ref = Good)								
Excellent	**0.75**	**[0.63, 0.89]**	0.80	[0.63, 1.03]	0.60	[0.47, 0.78]	0.71	[0.56, 0.91]
Fair	**1.42**	**[1.23, 1.64]**	**1.46**	**[1.21, 1.77]**	1.26	[0.99, 1.63]	1.28	[1.03, 1.61]
Poor	**1.86**	**[1.46, 2.39]**	**2.06**	**[1.52, 2.83]**	1.36	[0.89, 2.13]	1.47	[1.00, 2.20]
Psychological distress (Ref = Presence)								
Absence	**0.15**	**[0.13, 0.17]**	**0.17**	**[0.14, 0.20]**	**0.15**	**[0.13, 0.18]**	**0.17**	**[0.14, 0.20]**
Addiction (Ref = Presence)								
Absence	0.78	[0.66, 0.93]	0.90	[0.71, 1.14]	0.69	[0.54, 0.87]	0.73	[0.57, 0.93]
Informal therapeutic strategies (e.g. yoga; Ref = Use)								
Non-use	**0.63**	**[0.54, 0.74]**	**0.59**	**[0.47, 0.73]**	**0.67**	**[0.54, 0.84]**	**0.73**	**[0.58, 0.90]**
OTC medication (Ref = Use)								
Non-use	0.78	[0.64, 0.95]	0.66	[0.50, 0.87]	0.90	[0.98, 1.18]	0.90	[0.68, 1.18]
Self-medication with alcohol/drugs (Ref = Use)								
Non-use	0.92	[0.80, 1.06]	0.86	[0.71, 1.04]	1.04	[0.85, 1.27]	1.15	[0.94, 1.40]
Non-clinical care (e.g. helplines; Ref = Use)								
Non-use		N/A		N/A		N/A	**0.31**	**[0.18, 0.48]**
Interpersonal support (e.g. family; Ref = Use)								
Non-use		N/A		N/A		N/A	0.81	[0.66, 0.99]
Number of self-harm methods used (0–6)		N/A		N/A		N/A	**1.37**	**[1.17, 1.62]**

Two-level hierarchical models were performed for the self-harm subgroup. This is because certain self-harm-specific variables (i.e. non-clinical care for self-harm, interpersonal support for self-harm, and number of self-harm methods used) were only available for those reporting self-harm behaviours. In the total sample and the SI only subgroup, self-harm-related variables were not included in the multivariable regression model, as a large proportion of participants did not self-harm. In the SI with SA subgroup, the absence/presence of a mental health diagnosis was not tested in the planned analysis due to insufficient observations in some categorical levels, and thus, no sensitivity analysis excluding the absence/presence of a mental health diagnosis was conducted for the SI with SA subgroup. No regression model was run for the Death by Suicide subgroup due to insufficient data. Bold values denote significance based on a Bonferroni-corrected *P* threshold of 0.002.

When examining these factors within specific suicidality subgroups:

In the SI only subgroup, male sex, absence of psychological distress, non-use of informal therapeutic strategies, good perceived overall health (vs. fair and vs. poor) and living with a partner (vs. living alone), were associated with reduced odds of treatment receipt.

In the self-harm subgroup, the base model showed that male sex, absence of psychological distress, non-use of informal therapeutic strategies and absence of confiding were associated with reduced odds of treatment receipt. In the full model, male sex, absence of psychological distress and absence of confiding remained linked to reduced odds of treatment receipt, while non-use of informal therapeutic strategies did not. Additionally, non-use of helplines and using fewer types of self-harm methods were linked to reduced odds of treatment receipt.

### Factors associated with treatment seeking and receipt in those with NSSI vs. SSI

Data insufficiencies/imbalances prevented a comprehensive analysis of the factors associated with treatment seeking and receipt in the SI with SA and Death by Suicide subgroups. Therefore, we sought to supplement our primary analyses with exploratory analyses evaluating factors associated with treatment seeking and receipt across groups with different types of self-harm behaviours. Specifically, we focused on those with NSSI and those with SSI. When examined alongside the SI Only subgroup, we hypothesised that these groups would represent increasing levels of suicide risk, from NSSI to SI Only to SSI, as evidence indicates that SSI is a greater risk factor for suicide compared to SI^30^, which poses greater risk than NSSI^31^. This allowed us to examine whether correlates of treatment seeking and receipt differ as a function of suicidality severity. To do this, the self-harm subgroup was divided into SSI and NSSI subgroups based on responses to the MHQ items assessing suicidal ideation and past suicide attempts (see Supplementary Materials for further detail), and regression models were run separately within the SSI and NSSI subgroups. As in the primary analysis, we ran two models where the base model included all explanatory factors, except for the absence/presence of a mental health diagnosis and self-harm-specific variables. Those self-harm-specific variables were then added to the full model in the second step. Forest plots displaying odds ratios and 95% confidence intervals for the associations between each factor and treatment seeking and receipt for the NSSI, SI Only, and SSI subgroups are shown in Supplementary Fig. 1.

In the NSSI subgroup, the base model revealed that male sex, absence of psychological distress, and non-use of informal therapeutic strategies were associated with reduced odds of treatment seeking, while male sex and absence of psychological distress were associated with reduced odds of treatment receipt. In the full model, with the addition of self-harm-specific variables, those associations remained, without any additional factors identified.

In the SSI subgroup, in the base model, male sex and absence of psychological distress were associated with reduced odds of treatment seeking and reduced odds of treatment receipt. In the full model, these associations remained, with two additional factors—non-use of helplines and use of fewer methods of self-harm—emerging as associated with reduced odds of both treatment seeking and treatment receipt.

A complete summary of significant factors associated with reduced odds of treatment seeking and receipt across all participants and within the different subgroups is provided in Table 3.

**Table 3:** Summary of factors significantly associated with reduced treatment-related behaviours in the primary analyses for the total sample, SI only, self-harm, NSSI and SSI subgroups

Outcome	All participants	SI only	Self-harm					
			Entire Self-harm subgroup		NSSI		SSI	
			Base model	Full model	Base model	Full model	Base model	Full model
Reduced odds of treatment seeking (MHQ, 2016)	Male sex	Male sex	Male sex	Male sex	Male sex	Male sex	Male sex	Male sex
	Absence of psychological distress	Absence of psychological distress	Absence of psychological distress	Absence of psychological distress	Absence of psychological distress	Absence of psychological distress	Absence of psychological distress	Absence of psychological distress
	Non-use of informal therapeutic strategies	Non-use of informal therapeutic strategies	Non-use of informal therapeutic strategies	Non-use of informal therapeutic strategies	Non-use of informal therapeutic strategies	Non-use of informal therapeutic strategies		
	Good vs fair, vs poor perceived health	Good vs fair, vs poor perceived health	Excellent vs good perceived health	Excellent vs good perceived health				
				Non-use of non-clinical care				Non-use of non-clinical care
								Fewer self-harm methods
	Absence of confiding							
	Ethnic minority							
Reduced odds of treatment receipt (MHQ, 2016)	Male sex	Male sex	Male sex	Male sex	Male sex	Male sex	Male sex	Male sex
	Absence of psychological distress	Absence of psychological distress	Absence of psychological distress	Absence of psychological distress	Absence of psychological distress	Absence of psychological distress	Absence of psychological distress	Absence of psychological distress
	Non-use of informal therapeutic strategies	Non-use of informal therapeutic strategies	Non-use of informal therapeutic strategies					
	Good vs fair, good vs poor, excellent vs good perceived health	Good vs fair, vs poor perceived health						
		Living with a partner						
	Absence of confiding		Absence of confiding	Absence of confiding				
				Non-use of non-clinical care				Non-use of non-clinical care
				Fewer self-harm methods				Fewer self-harm methods
	Ethnic minority							

### Change in treatment-related behaviours

The primary cross-sectional analyses identified several factors linked to reduced odds of treatment seeking and receipt, including male sex, absence of psychological distress, non-use of informal therapeutic strategies, lower perceived overall health, living with a partner, absence of confiding, minority ethnicity, non-use of helplines and fewer self-harm methods. To investigate whether any of these factors might play a causal role in treatment-related behaviours, we conducted longitudinal analyses to examine factors that predicted a lack of change in treatment seeking and receipt over a six-year period among individuals who initially reported no treatment seeking or receipt, using data collected in 2016 and 2022. Further details are provided in Supplementary Materials.

Absence of psychological distress (OR = 1.58, 95% CI [1.18, 2.12]) and non-use of informal therapeutic strategies (OR = 1.82, 95% CI [1.28, 2.61]) predicted a lack of change from no treatment seeking in 2016 to treatment seeking in 2022. Absence of psychological distress (OR = 1.78, 95% CI [1.35, 2.32]) was the only predictor of a lack of change from no treatment receipt in 2016 to treatment receipt in 2022.

### Predictors of use of informal help vs. treatment seeking vs treatment receipt

In addition to information about formal treatment seeking and receipt, data on the use of informal support services (operationalised as the use of helplines) was also available for individuals reporting self-harm behaviours in the UKB dataset. This enabled us to examine whether factors predictive of treatment seeking and receipt in this subgroup reflect factors that may be predictive of help-seeking more generally (whether this be formal or informal), or whether different factors may be linked to use of informal versus formal support.

**Fig. 2:**
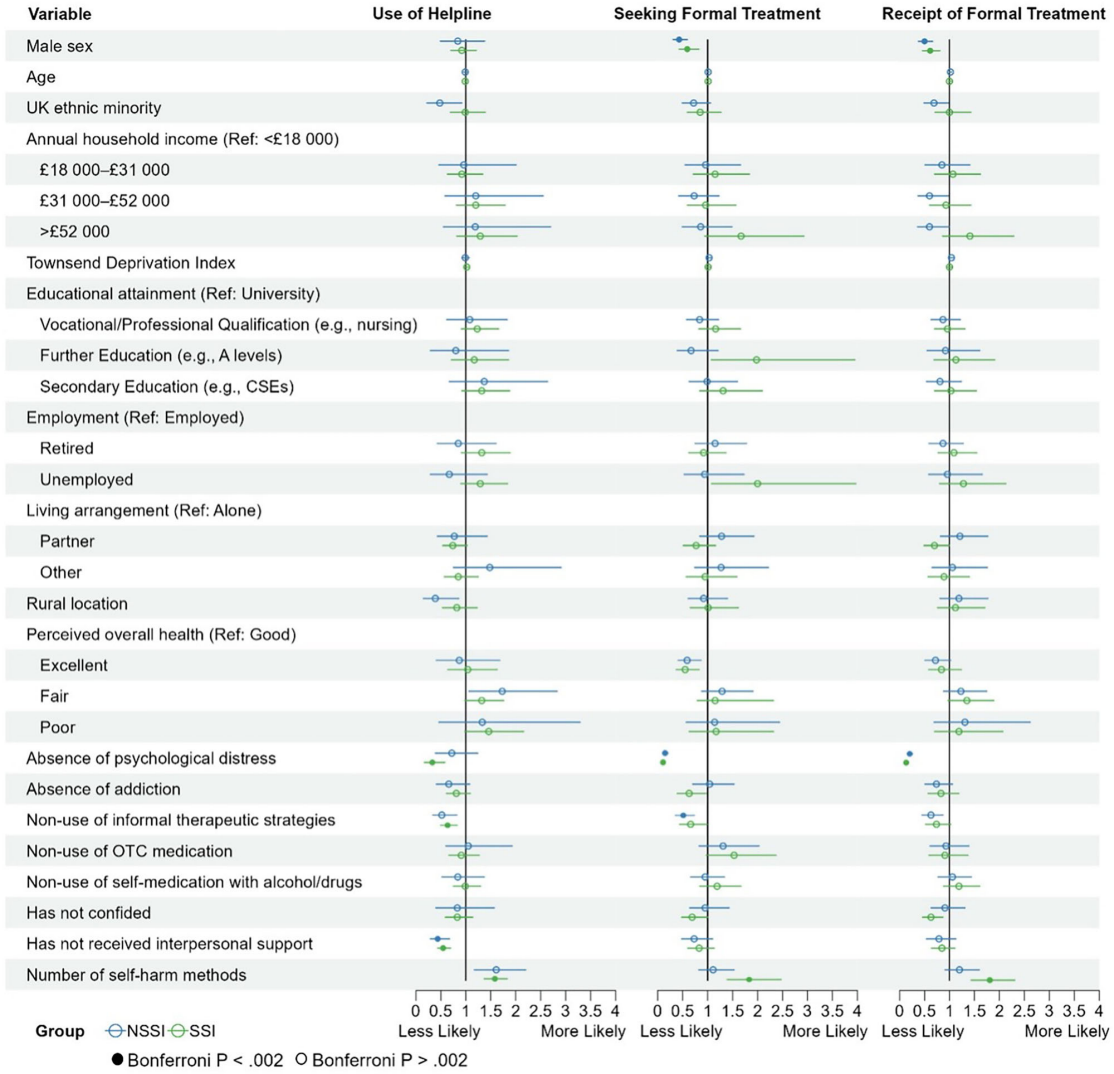
Factors associated with helpline use and formal treatment receipt in the NSSI and SSI subgroups. Forest plots showing the odds ratios (with 95% confidence intervals) for each factor in the multivariable models, examining their associations with use of helpline, seeking formal treatment, and receipt of formal treatment, respectively, across the non-suicidal self-injury (NSSI) and suicidal self-injury (SSI) subgroups. Figure symbols: Blue circles = NSSI subgroup; green circles = SSI subgroup; filled in circles = significant at Bonferroni-corrected threshold (*P* < 0.002); empty circles = not significant at Bonferroni-corrected threshold (i.e. *P* > 0.002).

Forest plots displaying odds ratios and 95% confidence intervals for these associations are shown in Fig. 2, with detailed results reported in the Supplementary Materials. Several factors that we had previously found were associated with reduced odds of seeking or receiving formal treatment were not associated with the odds of using informal support. Specifically, although male sex was consistently associated with reduced odds of formal treatment seeking and receipt, it was not linked to reduced odds of using informal support. Furthermore, several factors were significantly associated with the use of informal support that were not associated with formal treatment seeking or receipt. Factors specifically linked to reduced use of informal support included absence of interpersonal support, living with a partner and non-use of informal therapeutic strategies.

## Discussion

This study is the first to evaluate factors associated with treatment-related behaviours across varying levels of suicidality, using large-scale data. Results show that male sex, absence of psychological distress and non-use of informal support correlate with decreased treatment seeking and receipt across suicide risk levels. Some factors varied by suicide risk level; for example, living with a partner reduced the likelihood of treatment receipt (not seeking) specifically in the SI only subgroup. Although use of informal support was an important correlate of formal treatment seeking/receipt across groups, certain types of informal support were more relevant to formal treatment use in some groups than others. Specifically, use of informal therapeutic strategies (e.g. yoga or art classes) was most strongly correlated with formal service use among lower-risk individuals (SI only and NSSI subgroups), whereas use of helplines was most strongly correlated with formal service use among higher-risk individuals (SSI subgroup). Some factors also varied by type of treatment-related behaviours. For example, use of informal therapeutic strategies was associated with treatment seeking but not receipt in the NSSI subgroup. The results of longitudinal analyses confirmed a link between use of informal support and future use of formal services. These findings suggest that factors associated with treatment-related behaviours vary by suicide risk level and service engagement stage, and highlight the importance of incorporating the use of informal support into the service use pathway as a potential means of enhancing formal service utilisation.

Our findings indicate that individuals experiencing suicidality who do not engage with formal services exhibit distinct sociodemographic profiles, with male sex and living arrangement correlating with reduced treatment-related behaviours in the UKB sample. This supports existing research showing lower treatment rates among males experiencing suicidality^14,16,18,19,32,33^. Additionally, while some prior studies also suggest that living with a partner may deter service use^34,35^, others contradict this^36^, with mixed results likely stemming from a prior focus on suicide decedents^34–36^. Our study adds to the literature by showing that for individuals experiencing suicidal ideation, living with a partner may hinder receipt of formal treatment. Further research is needed to understand why this occurs, particularly regarding treatment barriers that may be intensified for those who cohabitate, such as treatment cost, perceptions of stigma, and reliance on a partner for support.

Our findings indicate that individuals with less severe suicidality are less likely to use formal services. Specifically, the SI only subgroup was less likely to seek and receive treatment relative to the SI with SA subgroup. Additionally, among individuals with past suicide attempts, those who used fewer self-harm methods were less likely to seek and receive treatment compared to those who used a greater variety of methods, which in prior research, has been linked to increased suicide risk^37^. These findings align with numerous studies suggesting that individuals at lower suicide risk are less likely to be in contact with mental health services^17,19,21,23,38,39^. Given that suicidal thoughts can rapidly transition to suicidal behaviours^40,41^, and a history of suicide attempts is one of the strongest predictors of death by suicide^42^, our findings emphasise the importance of early identification of suicide risk (e.g. through GP screening) and early intervention to manage suicidal thoughts and behaviours^43^.

Other mental health indicators—such as the absence of a mental health disorder diagnosis and lack of psychological distress—were consistently associated with lower rates of treatment seeking and receipt across levels of suicidality. These factors have been frequently associated with non-use of services among individuals experiencing suicidal thoughts or behaviours^14–18,22,23,39,44^, and those who died by suicide^14,16,21^. Longitudinal analysis showed that individuals who disengaged from services were more likely to remain out of contact if they did not experience psychological distress, suggesting that the absence of distress may play a role in long-term service non-use. Interestingly, over 30% of individuals experiencing current suicidal ideation reported not feeling distressed, suggesting that suicidal thoughts can be quite diverse. Some individuals may experience acute ideation triggered by situational stressors (e.g. relationship breakdown), while others may have chronic, intermittent suicidal thoughts not linked to specific triggers^45^. The latter group may not perceive a need for help, reducing their likelihood of seeking formal services.

Seeking support from informal sources is common among individuals experiencing suicidality and self-harm, and is widely accepted as a gateway to mental health services^46–51^. Our findings reiterate the notion that use of informal support is associated with engagement with formal services, but extend existing work by showing that the type of informal support most strongly linked to formal service use varies by suicidality level. The difference in associations may reflect the different needs and preferences of those at varying risk levels, as well as the distinct ways in which different types of informal supports feed into formal services based on an individual’s level of risk. For example, individuals at higher levels of risk may prefer the more immediate and personalised support offered via helplines over other informal therapeutic strategies, and furthermore, when higher-risk individuals do use helplines, these helplines may be more likely to refer them into formal care, given the heightened perceptions of risk. In contrast, helplines may be less likely to refer lower-risk individuals into formal care and may instead recommend other strategies for self-management. We further found that non-use of informal therapeutic strategies was linked to long-term disengagement from services, indicating that non-use of informal support may hinder future contact with professional services.

An alternative explanation is that a third variable is driving the association between use of helplines and use of formal services among higher-risk individuals—e.g. a general propensity towards disclosure or help-seeking. To assess this possibility, we looked at whether the predictors of use of formal services and use of informal services (e.g. helplines) were the same or different. While the absence of interpersonal support was uniquely linked to non-use of helplines (but not non-use of formal services), several common predictors emerged, including the absence of psychological distress, fewer self-harm methods and non-use of informal therapeutic strategies. These findings indicate that experience of distress, greater risk of suicide, and a general propensity towards help-seeking may simultaneously be associated with engagement with both formal and informal services. Additionally, it is possible that some unobserved variables, such as stigma surrounding suicidality and/or help seeking, low mental health literacy, and high self-reliance—commonly identified as barriers to help-seeking among men in prior literature^20^—could also deter use of both formal and informal services.

Several limitations should be noted. First, some previously identified barriers to formal care were not captured by the UKB, including personality characteristics (e.g. detachment)^15^, psychological factors (e.g. entrapment, self-reliance and stigma)^15,46,52^, and experience with formal services (e.g. prior use, satisfaction and perceived effectiveness)^46,52^. Incorporating those established predictors into future research could deepen our understanding of the distinct profiles of at-risk individuals with different rates of service use. Second, like many previous studies^15,17–19,22^, the UKB measured treatment seeking and receipt for general mental health problems such as anxiety and depression, not specifically for suicidality, potentially leading to an overestimation of the reported utilisation of formal services for suicidality. Additionally, details of treatment engagement—such as the frequency, time frame, and duration of those behaviours—were not captured, limiting the understanding of the dynamic process of service contact. Further, the UKB item used to define suicidal ideation captures passive ideation. While this item has been applied in prior research as a measure of suicidal ideation^27,53^ and is drawn from the Patient Health Questionnaire-9, which is commonly used in clinical practice for suicide risk screening, active ideation is conceptually and clinically distinct from passive ideation. Future research should incorporate measures of active suicidal ideation to better capture the full spectrum of suicidal thoughts and more precisely identify those at elevated risk. Additionally, when determining death by suicide, we excluded individuals with undetermined intent deaths who did not report prior suicidality to ensure rigorous classification. Yet, factors such as stigma, lack of disclosure, or missing records may obscure the true rates of suicide deaths, highlighting the need to improve documentation and classification of mortality data. Finally, the UKB sample’s older age range (e.g. 40–70 years), predominantly White British ethnicity, and lack of data on gender diversity may limit the generalisability of findings to broader, more diverse populations.

The current findings have important clinical implications. Although many suicide prevention efforts have been implemented to promote service usage, such as gatekeeper training and psychoeducation^54^, existing mental health care systems may not adequately meet the unique needs and preferences of specific subgroups experiencing suicidality, such as individuals not feeling distressed or not using alternative support. Suicide prevention initiatives across a broader range of settings, such as screening-based programs and gatekeeper training in the communities, workplaces, schools/universities, and primary care settings, or digital tools for real-time risk monitoring and tailored support^55^, are needed to link those without severe mental health problems to appropriate care. Mental health service usage may also benefit from informal support strategies that target specific subgroups. For example, promoting informal therapeutic activities for those experiencing suicidal thoughts, and crisis helplines for those with prior suicide attempts, could be particularly effective.

## Supplementary information

Supplementary Materials_Tang_etal.

## References

[1] Ilic, M. & Ilic, I. Worldwide suicide mortality trends (2000-2019): a joinpoint regression analysis. *World J. Psychiatry* **12**, 1044–1060 (2022). 36158305 10.5498/wjp.v12.i8.1044 PMC9476842

[2] Naghavi, M. Global, regional, and national burden of suicide mortality 1990 to 2016: systematic analysis for the Global Burden of Disease Study 2016. *BMJ* **364**, l94 (2019). 31339847 10.1136/bmj.l94 PMC6598639

[3] Statistics, O. f. N. Suicides in England and Wales: 2022 registrations. *Office for National Statistics* (2023).

[4] Hofstra, E. et al. Effectiveness of suicide prevention interventions: a systematic review and meta-analysis. *Gen. Hosp. Psychiatry* **63**, 127–140 (2020). 31078311 10.1016/j.genhosppsych.2019.04.011

[5] Méndez-Bustos, P. et al. Effectiveness of psychotherapy on suicidal risk: a systematic review of observational studies. *Front. Psychol.* **10**, 277 (2019). 10.3389/fpsyg.2019.00277 PMC6389707 30837920

[6] Sufrate-Sorzano, T. et al. Interventions of choice for the prevention and treatment of suicidal behaviours: an umbrella review. *Nurs. Open* **10**, 4959–4970 (2023). 37218123 10.1002/nop2.1820 PMC10333855

[7] Cantor, J. H., McBain, R. K., Ho, P.-C., Bravata, D. M. & Whaley, C. Telehealth and in-person mental health service utilization and spending, 2019 to 2022. *JAMA Health Forum* **4**, e232645–e232645 (2023). 37624614 10.1001/jamahealthforum.2023.2645 PMC10457709

[8] Kazdin, A. E. Annual research review: expanding mental health services through novel models of intervention delivery. *J. Child Psychol. Psychiatry* **60**, 455–472 (2019). 29900543 10.1111/jcpp.12937

[9] Bruffaerts, R. et al. Treatment of suicidal people around the world. *Br. J. Psychiatry* **199**, 64–70 (2011). 21263012 10.1192/bjp.bp.110.084129 PMC3167419

[10] Carter, G. et al. Characteristics of suicide decedents with no federally funded mental health service contact in the 12 months before death in a population-based sample of Australians 45 years of age and over. *Suicide Life Threat. Behav.* **53**, 110–123 (2023). 36353997 10.1111/sltb.12928 PMC10947544

[11] Hom, M. A., Stanley, I. H. & Joiner, T. E. Jr. Evaluating factors and interventions that influence help-seeking and mental health service utilization among suicidal individuals: a review of the literature. *Clin. Psychol. Rev.* **40**, 28–39 (2015). 26048165 10.1016/j.cpr.2015.05.006

[12] Janota, M., Kovess-Masfety, V., Gobin-Bourdet, C. & Husky, M. M. Use of mental health services and perceived barriers to access services among college students with suicidal ideation. *J. Behav. Cogn. Ther.* **32**, 183–196 (2022).

[13] Stene-Larsen, K. & Reneflot, A. Contact with primary and mental health care prior to suicide: a systematic review of the literature from 2000 to 2017. *Scand. J. Public Health* **47**, 9–17 (2019). 29207932 10.1177/1403494817746274

[14] Youn, H. M., Kang, S. H., Jang, S.-I. & Park, E.-C. Association between social participation and mental health consultation in individuals with suicidal ideation: a cross-sectional study. *BMC Psychiatry* **20**, 305 (2020). 32546143 10.1186/s12888-020-02724-8 PMC7296757

[15] Batterham, P. J. et al. Factors associated with professional mental health service use among adults with suicidal ideation. *J. Affect. Disord.* **307**, 278–285 (2022). 35398106 10.1016/j.jad.2022.04.013

[16] Han, J., Batterham, P. J., Calear, A. L. & Randall, R. Factors influencing professional help-seeking for suicidality. *Crisis* **39**, 175–196 (2018). 29052431 10.1027/0227-5910/a000485

[17] Islam, M. I., Khanam, R. & Kabir, E. The use of mental health services by Australian adolescents with mental disorders and suicidality: Findings from a nationwide cross-sectional survey. *PLoS ONE* **15**, e0231180 (2020). 32275704 10.1371/journal.pone.0231180 PMC7147749

[18] Milner, A. & De Leo, D. Who seeks treatment where? Suicidal behaviors and health care: evidence from a community survey. *J. Nerv. Ment. Dis.* **198**, 412–419 (2010). 20531119 10.1097/NMD.0b013e3181e07905

[19] Mok, K. et al. Factors associated with help-seeking for emotional or mental health problems in community members at risk of suicide. *Adv. Mental Health* **19**, 236–246 (2021).

[20] Reily, N. M. et al. Help-seeking and barriers to service use amongst men with past-year suicidal ideation and not in contact with mental health services. *Arch. Suicide Res.* **28**, 482–498 (2024). 36987997 10.1080/13811118.2023.2190781

[21] Tang, S. et al. People who die by suicide without receiving mental health services: a systematic review. *Front. Public Health* **9**, 736948 (2022b). 10.3389/fpubh.2021.736948 PMC8804173 35118036

[22] Tang, S. et al. Predictors of not receiving mental health services among people at risk of suicide: a systematic review. *J. Affect. Disord.* **301**, 172–188 (2022a). 10.1016/j.jad.2022.01.054 35032506

[23] Tang, S. et al. Correlates of non-receipt of formal mental health services among Australian men experiencing thoughts of suicide. *J. Affect. Disord. Rep.* **11**, 100455 (2023).

[24] Law, Y. -w, Wong, P. W. C. & Yip, P. S. F. Suicide with psychiatric diagnosis and without utilization of psychiatric service. *BMC Public Health* **10**, 431 (2010). 20649996 10.1186/1471-2458-10-431 PMC2920278

[25] Giacco, D. & Priebe, S. Suicidality and hostility following involuntary hospital treatment. *PLoS ONE* **11**, e0154458 (2016). 27171229 10.1371/journal.pone.0154458 PMC4865189

[26] Klonsky, E. D., Saffer, B. Y. & Bryan, C. J. Ideation-to-action theories of suicide: a conceptual and empirical update. *Curr. Opin. Psychol.* **22**, 38–43 (2018). 30122276 10.1016/j.copsyc.2017.07.020

[27] Na, P. J. et al. Psychosocial moderators of polygenic risk for suicidal ideation: results from a 7-year population-based, prospective cohort study of U.S. veterans. *Mol. Psychiatry* **27**, 1068–1074 (2022). 34725455 10.1038/s41380-021-01352-2

[28] Rayner, C. et al. Genetic influences on treatment-seeking for common mental health problems in the UK biobank. *Behav. Res. Ther.* **121**, 103413 (2019). 31491689 10.1016/j.brat.2019.103413 PMC6873796

[29] Ruderfer, D. M. et al. Significant shared heritability underlies suicide attempt and clinically predicted probability of attempting suicide. *Mol. Psychiatry* **25**, 2422–2430 (2020). 30610202 10.1038/s41380-018-0326-8 PMC6609505

[30] Hyman, J., Ireland, R., Frost, L. & Cottrell, L. Suicide incidence and risk factors in an active duty US military population. *Am. J. Public Health* **102**, S138–S146 (2012). 22390588 10.2105/AJPH.2011.300484 PMC3496445

[31] Wichstrøm, L. Predictors of non-suicidal self-injury versus attempted suicide: similar or different?. *Arch. Suicide Res.* **13**, 105–122 (2009). 19363748 10.1080/13811110902834992

[32] De Leo, D., Cerin, E., Spathonis, K. & Burgis, S. Lifetime risk of suicide ideation and attempts in an Australian community: prevalence, suicidal process, and help-seeking behaviour. *J. Affect. Disord.* **86**, 215–224 (2005). 15935241 10.1016/j.jad.2005.02.001

[33] Wong, J., Brownson, C., Rutkowski, L., Nguyen, C. P. & Becker, M. S. A mediation model of professional psychological help seeking for suicide ideation among Asian American and white American college students. *Arch. Suicide Res.* **18**, 259–273 (2014). 24620900 10.1080/13811118.2013.824831

[34] Hamdi, E., Price, S., Qassem, T., Amin, Y. & Jones, D. Suicides not in contact with mental health services: risk indicators and determinants of referral. *J. Mental Health* **17**, 398–409 (2008).

[35] Schaffer, A. et al. Population-based analysis of health care contacts among suicide decedents: identifying opportunities for more targeted suicide prevention strategies. *World Psychiatry* **15**, 135–145 (2016). 27265704 10.1002/wps.20321 PMC4911782

[36] Salib, E. & Green, L. Gender in elderly suicide: analysis of coroners inquests of 200 cases of elderly suicide in Cheshire 1989-2001. *Int. J. Geriatr. Psychiatry* **18**, 1082–1087 (2003). 14677139 10.1002/gps.1012

[37] Zahl, D. L. & Hawton, K. Repetition of deliberate self-harm and subsequent suicide risk: long-term follow-up study of 11,583 patients. *Br. J. Psychiatry* **185**, 70–75 (2004). 15231558 10.1192/bjp.185.1.70

[38] Ahmedani, B. K. et al. Suicide thoughts and attempts and psychiatric treatment utilization: informing prevention strategies. *Psychiatr. Serv.* **63**, 186–189 (2012). 22302340 10.1176/appi.ps.201100159 PMC3281500

[39] Wang, X. et al. Suicide risk help-seeking among middle- to old-age adults: a systematic review. *Innov. Aging* **7**, igac079 (2023). 36815014 10.1093/geroni/igac079 PMC9940623

[40] Deisenhammer, E. A. et al. The duration of the suicidal process: how much time is left for intervention between consideration and accomplishment of a suicide attempt?. *J. Clin. Psychiatry* **70**, 19–24 (2009). 19026258

[41] Sunderland, M., Batterham, P. J., Calear, A. L., Chapman, C. & Slade, T. Factors associated with the time to transition from suicidal ideation to suicide plans and attempts in the Australian general population. *Psychol. Med.* **53**, 258–266 (2023). 33926588 10.1017/S0033291721001501

[42] Bostwick, M., Pabbati, C., Geske, J. R. & McKean, A. J. Suicide attempt as a risk factor for completed suicide: even more lethal than we knew. *Am. J. Psychiatry* **173**, 1094–1100 (2016). 27523496 10.1176/appi.ajp.2016.15070854 PMC5510596

[43] Granö, N. et al. Declines in suicidal ideation in adolescents being treated in early intervention service. *Psychosis* **8**, 176–179 (2016).

[44] Bobevski, I., Rosen, A. & Meadows, G. Mental health service use and need for care of Australians without diagnoses of mental disorders: findings from a large epidemiological survey. *Epidemiol. Psychiatr. Sci.* **26**, 596–606 (2017). 28625212 10.1017/S2045796017000300 PMC6998986

[45] Sivak, J., Swartz, J. L. & Swenson, D. X. PTSD And chronic suicidal ideation:the role of counter suicidal cognition. *Traumatology* **5**, 1–6 (1999).

[46] Arria, A. M. et al. Help seeking and mental health service utilization among college students with a history of suicide ideation. *Psychiatr. Serv.* **62**, 1510–1513 (2011). 22193801 10.1176/appi.ps.005562010 PMC3246367

[47] Brownson, C., Drum, D. J., Smith, S. E. & Burton Denmark, A. Differences in suicidal experiences of male and female undergraduate and graduate students. *J. Coll. Stud. Psychother.* **25**, 277–294 (2011).

[48] Michelmore, L. & Hindley, P. Help-seeking for suicidal thoughts and self-harm in young people: a systematic review. *Suicide Life Threat. Behav.* **42**, 507–524 (2012). 22889130 10.1111/j.1943-278X.2012.00108.x

[49] Nguyen, M.-H. et al. Alice in Suicideland: exploring the suicidal ideation mechanism through the sense of connectedness and help-seeking behaviors. *Int. J. Environ. Res. Public Health* **18**, 3681 (2021). 33916123 10.3390/ijerph18073681 PMC8037954

[50] Rowe, S. L. et al. Help-seeking behaviour and adolescent self-harm: a systematic review. *Aust. N. Z. J. Psychiatry* **48**, 1083–1095 (2014). 25335872 10.1177/0004867414555718

[51] Calear, A. L. & Batterham, P. J. Suicidal ideation disclosure: Patterns, correlates and outcome. *Psychiatry Res.* **278**, 1–6 (2019). 31128420 10.1016/j.psychres.2019.05.024

[52] Czyz, E. K., Horwitz, A. G., Eisenberg, D., Kramer, A. & King, C. A. Self-reported barriers to professional help seeking among college students at elevated risk for suicide. *J. Am. Coll. Health* **61**, 398–406 (2013). 24010494 10.1080/07448481.2013.820731 PMC3788673

[53] Harrison, R., Munafò, M. R., Smith, G. D. & Wootton, R. E. Examining the effect of smoking on suicidal ideation and attempts: a triangulation of epidemiological approaches. *Br J Psychiatry* **217**, 701–707 (2020). 10.1192/bjp.2020.68 PMC7705667 32290872

[54] Robinson, J. et al. What works in youth suicide prevention? A systematic review and meta-analysis. *EClinicalMedicine* **4-5**, 52–91 (2018). 31193651 10.1016/j.eclinm.2018.10.004 PMC6537558

[55] Coppersmith, D. D. L. et al. Just-in-time adaptive interventions for suicide prevention: promise, challenges, and future directions. *Psychiatry* **85**, 317–333 (2022). 35848800 10.1080/00332747.2022.2092828 PMC9643598

