# Supplementary material for: Identifying distinct service use phenotypes across levels of suicidality and self-harm: a UK Biobank study
Source: Npj Ment Health Res. 2025 Sep 23;4:46. doi: 10.1038/s44184-025-00160-8 (PMC12457640; doi:10.1038/s44184-025-00160-8)
Supplement: Supplementary file 1 — Supplementary Materials_Tang_etal. [file 44184_2025_160_MOESM1_ESM.pdf]

Supplementary Information

Table of Contents

a) Categorisation and Recoding of Explanatory Variables

b) Statistical Analyses

c) Pre-registered Analyses

d) Factors Associated with Treatment-Related Behaviours in NSSI vs. SSI

e) Change in Treatment-Related Behaviours

f) Predictors of Change in Suicidality Severity Over Time

g) Factors Associated with Informal Help vs Treatment Seeking vs Treatment Receipt

h) Association Between Self-Harm Methods and Treatment-Related Behaviours

i) Table S1. UK Biobank items and corresponding field codes.

j) Table S2. Sample characteristics by subgroup and summarised results for group comparisons.

k) Table S3. Contingency table displaying the absence of a mental health diagnosis (vs. presence) in relation to treatment seeking as well as treatment receipt among all participants.

l) Table S4. Odds ratios and 95% confidence intervals from planned multivariable logistic regression models.

m) Figure S1. Forest plots showing the odds ratios (with 95% confidence intervals) for each factor in the multivariable models, examining their associations with treatment seeking and treatment receipt respectively, across the NSSI, SI Only and SSI subgroups.

n) Figure S2. Percentage of participants using each self-harm method.

o) Figure S3. Use of self-harm methods among treatment-seekers/non-seekers (A) and treatment receivers/non-receivers (B).

Categorisation and Recoding of Explanatory Variables

Sociodemographic Variables

Ethnic minority status was re-coded as 'white British' or 'ethnic minority', in line with prior research (Rayner et al., 2021). *Educational attainment* was re-coded into four categories: 'secondary', 'further', 'vocational', 'university degree'. *Employment status* was re-coded into three categories: 'employed' (either paid or self-employed), 'retired', or 'unemployed' (including unpaid/voluntary work, studying, looking after family, and inability to work due to sickness/disability). *Annual household income* was re-coded into four categories: '<£18,000', '£18,000–£31,000', '£31,000–£52,000', and '>£52,000' (due to the small proportion selecting >£100,000). The *Townsend Deprivation Index*, measured at recruitment, was used to capture neighbourhood deprivation, with positive scores indicating relative deprivation and negative scores indicating relative affluence. *Living arrangement* was derived from responses to questions about household size and relationships within the household and had three categories: 'living alone', 'living with a partner', or 'living with other'. *Location* was determined by home area population density, with responses re-categorised into 'rural' or 'urban'. *Emotional support* was assessed by the frequency of confiding in someone close, dichotomised into 'yes' or 'no'.

Health-Related Variables

Perceived overall health was assessed at baseline using responses to a self-rating scale and had four categories: 'excellent', 'good', 'fair', or 'poor'. All other health-related variables were assessed through the MHQ. *Mental health diagnosis* was determined based on participants' self-report of whether they had ever received a professional diagnosis of a mental health disorder, dichotomised into 'yes' or 'no'. *Addiction* was assessed via a question asking participants if they had ever experienced lifetime dependence or addiction to a

substance (excluding cigarettes/coffee) or behaviour (e.g., gambling), dichotomised into 'yes' or 'no'. The *number of self-harm methods used* was assessed by participants' self-reported methods of self-harm used, ranging from '0' to '6' (only available for the SI with SA and Self-harm subgroups).

Statistical Analyses

Prior to analysis, responses of "prefer not to answer" or "do not know" to any questions were coded as missing values. Potential differences in treatment-related behaviours and explanatory variables between the subgroups were examined using Chi-square or ANOVA tests.

To assess potential multicollinearity, a variance inflation factor (VIF) was calculated for each explanatory variable in the multivariate models. VIFs above 5 or 10 typically indicate multicollinearity. However, our results suggested no concerns for high correlation ($VIF_{max} = 1.21$).

Exploratory Analyses

Baseline Treatment Seeking. The first exploratory analysis examined the associations between the explanatory variables and baseline treatment seeking in the Death by Suicide subgroup. We decided to explore baseline treatment seeking due to insufficient MHQ completion by the Death by Suicide subgroup. Over 90% of participants in this subgroup did not respond to the MHQ questions assessing treatment seeking or receipt, as such no planned analyses could be conducted. Therefore, the assessment of treatment seeking at baseline might potentially provide valuable insights. Baseline treatment seeking was determined by participants' responses to two questions about if they had seen a psychiatrist of a GP for mental health problems. As baseline treatment seeking was assessed at baseline, when treated as an outcome, several exploratory variables had to be removed from the model

to ensure that the predictors did not precede the outcome, including mental health diagnosis, psychological distress, addiction, number of self-harm methods used and all help-related variables. For the same reason, the covariate symptom severity score was omitted for temporal consistency. Only a limited number of explanatory variables (sex, age, TDI, and confiding) were included in this model.

Sensitivity Analyses Excluding Mental Health Diagnosis. Due to an extremely strong relationship between the absence of a mental health disorder and non-seeking and non-receipt of treatment, sensitivity analyses excluding the absence/presence of a mental health disorder as a potential predictor were conducted as primary analyses for all participants and each subgroup, complementing the planned analyses. As self-harm-specific variables (use/non-use of helpline, presence/absence of interpersonal support, number of self-harm method) were only assessed in those reporting self-harm behaviours, but not those without prior self-harm behaviours, two nested models were performed for the Self-harm subgroup. All explanatory variables excluding self-harm-specific variables and absence/presence of a mental health disorder were included into the base model. In the full model, those self-harm-specific variables were added.

Change in Suicidality Severity. This exploratory analysis aimed to identify factors associated with the change in suicidality severity over time in the SI Only subgroup, assessed initially by the MHQ in 2016 and re-evaluated using the MWQ in 2022. Participants reporting no SI or SA in the MWQ was categorised as ‘improved’, those reporting SI but not SA as ‘no change’, and those reporting SA regardless of SI as ‘worsened’. Multivariable multinomial logistic regressions were then employed to identify factors associated with the six-year change in suicidality severity.

Change in Treatment Behaviours Over Time. We also explored changes in treatment-related behaviours from 2016 (assessed by MHQ) to 2022 (assessed by MWQ), specifically focusing on those who reported no treatment seeking or receipt in 2016. For individuals reporting no treatment seeking when asked in 2016, those remaining non-treatment-seekers in 2022 was categorised as 'no change' while those reporting treatment seeking in 2022 was categorised as 'improved'. The same was applied to treatment receipt. Multivariable logistic regressions were then employed to identify factors associated with the six-year change in treatment seeking and treatment receipt.

Self-Harm Subgroup and Self-Harm Methods. Given that the hospital inpatient records did not differentiate SSI and NSSI, this subgroup was divided into SSI and NSSI based on their self-reported responses to questions about SI and SA in the MHQ. To enhance accuracy, those who reported no SI or prior SA were categorised as NSSI (n=3083), while those reporting prior SA were categorised as SSI (n=3553). The reason that we incorporated the measure of SI in determining NSSI was because self-harm behaviours identified through hospital inpatient records post-MHQ completion may involve suicidal intentions, which the MHQ failed to capture. As such, excluding participants with suicidal thoughts at the time of the MHQ helps mitigate this possibility. We acknowledge potential limitations related to this categorisation, yet this would be the most stringent way to ensure those in the NSSI category had no intention to suicide when they self-harmed. As in the primary analysis, we ran two nested models where the base model included all explanatory factors, except for the absence/presence of a mental health diagnosis and self-harm-specific variables. Those self-harm-specific variables were then added in the full model. Further analyses were then conducted to explore total and individual self-harm method usage across sexes, and different treatment-related behaviours.

Pre-registered Analyses

For our planned analysis, we used multivariable logistic regression to identify factors associated with treatment seeking, treatment receipt and other treatment-related behaviours, measured on the Mental Health Questionnaire (MHQ; 2016 assessment). Regression models were first conducted across all participants, and then in each subgroup separately, including all explanatory variables as planned.

Treatment Seeking

Effect sizes (odds ratios) for each factor in the model predicting treatment seeking are reported in Supplementary Table 4. According to the primary analysis, across the entire sample, the following factors were linked to reduced odds of treatment seeking: being male (vs. female), absence (vs. presence) of a mental health diagnosis, absence (vs. presence) of psychological distress, and non-use (vs. use) of informal therapeutic strategies (e.g., yoga and art classes).

When examining these factors within specific suicidality subgroups:

SI Only Subgroup. Male sex, absence of a mental health diagnosis, absence of psychological distress, non-use of informal therapeutic strategies, were associated with reduced odds of treatment seeking in the SI Only subgroup.

SI with SA Subgroup. Due to a heavily imbalanced ratio of treatment seekers to non-seekers on the MHQ, regression models could not be run in the SI with SA subgroup.

Death by Suicide Subgroup. Due to insufficient sample size with treatment seeking data on the MHQ, regression models could not be run in the Death by Suicide subgroup. However, exploratory analysis within this subgroup was possible by using a separate measure

of treatment seeking – baseline treatment seeking - obtained at a different point in time, and with a more limited number of explanatory variables (sex, age, TDI, and confiding).

Baseline treatment seeking was determined by participants' responses to two questions asking them if they had seen a psychiatrist or a GP for mental health problems. Explanatory variables included in these analyses were restricted to those that were also administered at the baseline assessment (to ensure predictors did not precede the outcome) and those with sufficient observations in this subgroup, which were sex, age, TDI, and confiding. Male sex emerged as a significant predictor of reduced odds of treatment seeking (OR=.33, 95% CI [.18, .59]), consistent with the results of our planned analyses in the SI Only and Self-harm subgroups.

Self-Harm Subgroup. Similar factors were associated with reduced odds of treatment seeking as in the SI Only subgroup, namely, male sex, absence of a mental health diagnosis, absence of psychological distress, and non-use of helpline for self-harm (e.g., helplines).

Treatment Receipt

Effect sizes (odds ratios) for each factor in the model predicting treatment receipt are reported in Supplementary Table 4. According to the primary analysis, across the entire sample, similar factors were associated with reduced odds of treatment receipt as were associated with reduced odds of treatment seeking, namely, male sex, absence of a mental health diagnosis, absence of psychological distress, and non-use of informal therapeutic strategies. However, in contrast to treatment seeking, living with a partner (vs. living alone) was also associated with reduced odds of treatment receipt.

When examining these factors within specific suicidality subgroups:

SI Only Subgroup. Male sex, absence of a mental health diagnosis, absence of psychological distress, and living with a partner emerged as being associated with reduced odds of treatment receipt in the SI Only subgroup.

SI with SA Subgroup. Only a limited number of explanatory variables were included in this model: sex, age, ethnicity, income, TDI, living arrangement, perceived overall health, location, absence/presence of confiding, absence/presence of an addiction, non-use/use of self-medication, non-use/use of interpersonal support, number of self-harm methods. The remaining variables were removed due to insufficient observations within individual categories: educational attainment, employment status, absence/presence of a mental health diagnosis, absence/presence of psychological distress, non-use/use of informal therapeutic strategies, non-use/use of OTC medication, and non-use/use of helpline. Using fewer types of self-harm methods (e.g., ingesting excessive medication, self-injury) was the only factor significantly associated with reduced odds of treatment receipt in the SI with SA subgroup.

Death by Suicide Subgroup. No regression model could be run with treatment receipt as the outcome due to insufficient data in the Death by Suicide subgroup.

Self-harm Subgroup. Male sex, absence of a mental health diagnosis, absence of psychological distress, and non-use of helpline for self-harm were significantly associated with reduced odds of treatment receipt in the Self-harm subgroup.

Other Treatment-Related Behaviours

Supplementary Table 4 shows factors associated with other treatment-related behaviours (measured on the MHQ; defined in Supplementary Table 1). Across the entire sample, as observed with treatment seeking and receipt, male sex, absence of a mental health

diagnosis, absence of psychological distress, and non-use of informal therapeutic strategies were significantly associated with reduced odds of other treatment-related behaviours.

When examining these factors within specific suicidality subgroups:

SI Only Subgroup. The same four variables were also found to predict reduced odds of other treatment-related behaviours for the SI Only subgroup, including male sex, absence of a mental health diagnosis, absence of psychological distress, and non-use of informal therapeutic strategies.

SI with SA Subgroup. No regression model was conducted due to heavily imbalanced engagement vs non-engagement ratio (92.9% vs 7.1%) and insufficient observations in the non-engagement category.

Death by Suicide Subgroup. No regression model could be conducted due to insufficient data on other treatment-related behaviours.

Self-harm Subgroup. Absence of a mental health diagnosis, absence of psychological distress, non-use of informal therapeutic strategies, and non-use of helpline for self-harm were significantly associated with reduced odds of other treatment-related behaviours.

In addition to the MHQ, data on other treatment-related behaviours was also available at a second point in time, captured via the DHQ. This afforded an opportunity to test whether factors shown to be associated with other treatment-related behaviours at the first time point on the MHQ were also predictive of other treatment-related behaviours at the second time point on the DHQ. Among all participants, absence of a mental health diagnosis (OR=.03, 95% CI [.03, .04]), absence of psychological distress (OR=.40, 95% CI [.32, .51]), and non-use of informal therapeutic strategies (OR=.65, 95% CI [.51, .83]) were significantly associated with reduced odds of other treatment-related behaviours. In the SI Only and Self-

harm subgroups, absence of a mental health diagnosis (SI Only: OR=.04, 95% CI [.03, .04], Self-harm: OR=.04, 95% CI [.03, .05]) and absence of psychological distress (SI Only: OR=.40, 95% CI [.31, .50], Self-harm: OR=.35, 95% CI [.27, .44]) were also negatively associated with other treatment-related behaviours. In addition, male sex predicted reductions in other treatment-related behaviours in the Self-harm group (OR=.66, 95% CI [.52, .85]). Regression models could not be conducted for the SI with SA and Death by Suicide subgroups due to insufficient observations.

Sex-Stratified Analyses

Separate multivariable models were run to compare factors associated with treatment seeking, treatment receipt and other treatment-related behaviours in males and females for the planned analysis. Models were first run in males and females across the entire sample, and then also for males and females in each suicidality subgroup. Where an explanatory variable emerged as being significantly associated with treatment seeking, treatment receipt or other treatment-related behaviours in males but not in females (or vice versa), we used a two-sample z-test to assess whether there were significant sex differences in the effect size. Where a sex difference in effect size was present, further multivariable models that included any relevant variable × sex interaction term would have been conducted to confirm the moderating role of sex.

Results showed that although some of the explanatory variables were significantly associated with treatment seeking, treatment receipt or other treatment-related behaviours in females but not males (and vice versa), none of the corresponding independent-samples z-tests showed evidence of a significant difference in effect size between males and females. This indicates that sex did not moderate the association between any of the explanatory variables and treatment-related behaviours.

Factors Associated with Treatment-Related Behaviours in NSSI vs. SSI

This exploratory analysis aimed to identify factors associated with treatment-related behaviours among the Self-harm group as a function of whether suicidal intent was present or not. Briefly, among participants who were hospitalised due to self-harm, hospital inpatient records did not differentiate those who were admitted due to suicidal self-injury (SSI) from those who were admitted due to non-suicidal self-injury (NSSI). However, we used responses on the MHQ as a proxy measure for distinguishing those with SSI from those with NSSI. Specifically, among those in the Self-harm subgroup, we dichotomised participants into two groups: (1) a SSI subgroup (n=3083), comprising participants who had been hospitalised for self-harm and who also reported a prior SA in the MHQ (which we hypothesised may indicate the presence of suicidal intent); (2) a NSSI subgroup (n=3553), comprising participants who had been hospitalised for self-harm but who did not endorse SI or SA on the MHQ (which we hypothesised may indicate absence of suicidal intent). As in the primary analysis, we ran two nested models. The base model included all explanatory factors, except for the absence/presence of a mental health diagnosis and self-harm-specific variables. In the full models, those self-harm-specific variables were added as potential predictors.

Significant factors associated with treatment seeking and treatment receipt in the Self-harm subgroups with SSI and NSSI are shown in Table 3, and associated effect sizes summarised below.

Treatment Seeking

NSSI. The base model revealed that male sex (OR=.43, 95% CI [.32, .59]), absence of psychological distress (OR=.16, 95% CI [.12, .21]), and non-use of informal therapeutic strategies (OR=.49, 95% CI [.33, .70]) were significantly associated with reduced odds of

treatment seeking in the NSSI subgroup. Those associations remained in the full model, with no additional factor linked to reduced odds of treatment seeking.

SSI. Male sex (OR=.61, 95% CI [.44, .84]) and absence of psychological distress (OR=.11, 95% CI [.08, .15]) were significantly associated with reduced odds of treatment seeking in the SSI subgroup in the base model. In the full model, non-use of helpline (OR=.30, 95% CI [.14, .55]) and fewer self-harm methods (OR=.59, 95% CI [.46, .74]) were also linked to reduced odds of treatment seeking in addition to absence of psychological distress.

Treatment Receipt

NSSI. The base model revealed that male sex (OR=.48, 95% CI [.36, .65]), and absence of psychological distress (OR=.19, 95% CI [.15, .25]) were significantly associated with reduced odds of treatment seeking in the NSSI subgroup. No additional factors linked to reduced odds of treatment receipt were found after adding self-harm-specific variables as potential predictors in the full model.

SSI. The base model revealed that male sex (OR=.61, 95% CI [.46, .80]) and absence of psychological distress (OR=.52, 95% CI [.40, .68]) were significantly associated with reduced odds of treatment receipt in the SSI subgroup. Those associations remained in the full model, with non-use of helpline (OR=.30, 95% CI [.14, .55]) and fewer self-harm methods used (OR=.59, 95% CI [.46, .74]) emerging as additional factors linked to reduced odds of treatment receipt.

Change in Treatment-Related Behaviours

Among individuals who initially reported no treatment seeking in 2016, we examined factors associated with a lack of change to treatment seeking in 2022 (vs. a shift to treatment seeking in 2022). Since the sample size of this analysis is much smaller compared to the

primary analysis (n=1079 vs N=15966), we did not include ethnicity and confiding as potential predictors in this model because they were only found to be linked to treatment seeking in the full sample, but not in any subgroup. Although perceived overall health, use/non-use of helpline and number of self-harm methods were found to be linked to treatment seeking in at least one subgroup, they were also not included in the model due to insufficient observations within certain categories. As such, factors included in this model were sex, presence/absence of psychological distress and use/non-use of informal therapeutic strategies.

Similarly, among those who initially reported no treatment receipt in 2016, we examined factors associated with a lack of change to treatment receipt in 2022 (vs. a shift to treatment receipt in 2022). As with the analysis for the change in treatment seeking, ethnicity was not included as a potential predictor in this model because it was only found to be linked to treatment receipt in the full sample, but not in any subgroup. Although perceived overall health, use/non-use of helpline and number of self-harm methods were found to be linked to treatment receipt in at least one subgroup, they were also excluded as potential predictors due to insufficient observations within certain categories. As such, factors included in this model were sex, presence/absence of psychological distress, use/non-use of informal therapeutic strategies, presence/absence of confiding, and living arrangement.

Although a longitudinal analysis of predictors of future treatment-related behaviours was possible using data on treatment seeking and receipt measured by the MWQ in 2022, it was not performed. This is because the ratios of treatment seeking vs non-seeking and treatment receipt vs non-receipt remained largely unchanged between 2016 and 2022 (treatment seeking: 80.9% vs 82.2%, treatment receipt: 75.8% vs 79.3%), making such an analysis redundant with our primary cross-sectional analyses.

Predictors of Change in Suicidality Severity Over Time

This exploratory analysis aimed to identify factors associated with the change in suicidality severity in the SI Only subgroup from 2016 to 2022. For participants who reported SI on the MHQ in 2016, those who then reported no SI and no SA in the MWQ in 2022 were categorised as ‘improved’, those reporting SI but no SA were categorised as ‘no change’, and those reporting SA were categorised as ‘worsened’. Multivariable multinomial logistic regressions were then run to identify factors associated with the six-year change in suicidality severity. Sex was the only significant predictor of change in suicidality between 2016 to 2022, where female sex was associated with increased odds of worsening suicidality (i.e., going from SI Only in 2016 to SA by 2022), OR=2.22, 95% CI [1.45, 3. 42].

Factors Associated with Informal Help vs Treatment Seeking vs Treatment Receipt

As data for use/non-use of helpline were only available among individuals reporting self-harm behaviours, those models were only performed for the Self-harm subgroup, as well as the NSSI and SSI subgroups.

Self-Harm Subgroup

In the Self-harm subgroup, living with a partner (OR= .65, 95% CI [.50, .85]), absence of psychological distress (OR= .46, 95% CI [.31, .68]), non-use of informal therapeutic strategies (OR= .60, 95% CI [.49, .75]), absence of interpersonal support (OR= .46, 95% CI [.38, .57]), and fewer types of self-harm methods (OR= .62, 95% CI [.55, .70]) were associated with non-use of helpline (e.g., helplines). Male sex (OR=.53, 95% CI [.43, .65]; OR=.55, 95% CI [.56, .67]), absence of psychological distress (OR=.15, 95% CI [.12, .18]; OR=.16, 95% CI [.14, .20]), non-use of informal therapeutic strategies (OR=.56, 95% CI [.43, .73]; OR=.70, 95% CI [56, .87]), and fewer types of self-harm methods (OR=.74, 95% CI [.62, .89]; OR=.70, 95% CI [.59, .82]) were associated with lack

of treatment seeking and lack of treatment receipt. In addition, excellent perceived overall health (vs. good; OR= .58, 95% CI [.45, .75])) was also associated with lack of treatment seeking while absence of confiding (OR=.68, 95% CI [.55, .85]) was also associated with lack of treatment receipt.

SSI

Among individuals engaging in SSI behaviours, absence of psychological distress (OR= .32, 95% CI [.16, .57]), non-use of informal therapeutic strategies (OR= .63, 95% CI [.49, .83]), absence of interpersonal support (OR= .53, 95% CI [.42, .68]), and fewer types of self-harm methods (OR=.63, 95% CI [.55, .73]) were associated with non-use of helpline. Male sex (OR=.60, 95% CI [.44, .83]; OR=.60, 95% CI [.45, .79]), absence of psychological distress (OR= .12, 95% CI [.08, .16]; OR=.13, 95% CI [.10, .17]) and using fewer types of self-harm methods (OR=.55, 95% CI [.41, .72]; OR=.56, 95% CI [.44, .70]) were associated with lack of treatment seeking and lack of treatment receipt.

NSSI

Among those reporting NSSI behaviours, absence of interpersonal support (OR=.42, 95% CI [.27, .66]) was the only factor associated with non-use of helpline. Male sex (OR=.44, 95% CI [.32, .60]; OR=.49, 95% CI [.37, .65]) and absence of psychological distressed (OR=.16, 95% CI [.12, .22]; OR=.20, 95% CI [.15, .26]) were associated with lack of treatment seeking and lack of treatment receipt. Additionally, non-use of informal therapeutic strategies was also linked to lack of treatment seeking (OR=.51, 95% CI [.35, .73]).

Association Between Self-Harm Methods and Treatment-Related Behaviours

We conducted a final exploratory analysis to assess whether there was an association between use of different self-harm methods and reduced likelihood of treatment seeking

and/or receipt. The proportion of participants who endorsed using different self-harm methods is shown in Supplementary Figure 2. The most commonly reported self-harm methods were ingestion of excessive medication, followed by self-injury.

When comparing self-harm methods used by males and females, a higher proportion of females reported ingesting excessive medication compared to males $P<.001$. In contrast, a higher proportion of males reported using alcohol/drug ingestion, unlisted methods, stopping prescribed medication, and swallowing dangerous objects or products, compared to females (all *P*s<.001).

Usage of self-harm methods in treatment seekers/non-seekers and treatment receivers/non-receivers is shown in Supplementary Figure 3. Self-injury was significantly more prevalent among individuals who did not seek treatment ($P<.001$), but its usage did not differ by treatment receipt, $P=.084$. In contrast, self-harm methods that involve medication and/or substances, including ingesting excessive medication, ingesting alcohol/drug, and stopping prescribed medication were significantly more common among those who sought and received treatment compared to those who did not (all *P*s<.001). The use of unlisted methods and swallowing dangerous objects or products did not differ by treatment-related behaviours.

Supplementary Tables

Table S1. UK Biobank items and corresponding field codes: Detailed listing of UK Biobank items used for determining subgroups and assessing explanatory variables, and measuring outcome variable, along with their corresponding field codes, and item sources.

Variable	UKB Question	Field Code	Source
**Defining Subgroups**			
**Suicidal ideation (SI; assessed in 2016; planned and primary analyses)**	"Over the last 2 weeks, how often have you been bothered by any of the following problems? Thoughts that you would be better off dead or of hurting yourself in some way?"	F20513	MHQ
**Suicidal ideation (SI; assessed in 2022; exploratory longitudinal analyses)**	"Many people have thoughts that life is not worth living. Have you felt that way?"	F29108	MWQ
**Attempted suicide (SA; assessed in 2016; planned and primary analyses)**	"Have you harmed yourself with the intention to end your life?"	F20483	MHQ
**Attempted suicide (SA; assessed in 2022; exploratory longitudinal analyses)**	"Have you harmed yourself with the intention to end your life?"	F29116	MWQ
**Death by suicide**	Ascertained via linkage with the mortality data using ICD-10 codes X60-84 (intentional self-harm) and Y10-Y34 (undetermined intent), excluding Y33.0 (unspecified place). Cases coded under Y10-Y34 (undetermined intent) without a report of SI or SA in the MHQ were excluded from the death by suicide subgroup.	F40001	Mortality data

**Self-harm**	“Have you deliberately harmed yourself, whether or not you meant to end your life?”;	F20480,	MHQ
	Also ascertained through hospital admissions with a diagnosis of intentional self-harm (ICD-10 X60-X84, ICD-9 E950-958 [intentional self-harm]) and injury/poisoning of undetermined intent (ICD-10 Y10-34 excluding Y33.9, ICD-9 E980-989 excluding 988.8). Cases coded under Y10-Y34 or ICD-9 E980-E989 without self-reported self-harm in the MHQ were excluded from the self-harm subgroup.	F41270, F41271	Hospital inpatient data
**Treatment-Related Behaviours**			
**Treatment seeking (assessed in 2016; planned and primary analyses)**	"Did you ever tell a professional about these problems (medical doctor, psychologist, social worker, counsellor, nurse, clergy, or other helping professional)?" (Depression symptoms)	F20448	MHQ
	"Did you ever tell a professional about these problems (medical doctor, psychologist, social worker, counsellor, nurse, clergy, or other helping professional)?" (Anxiety symptoms)	F20428	MHQ
**Baseline treatment seeking (exploratory analysis)**	"Have you ever seen a psychiatrist for nerves, anxiety, tension or depression?"	F2100	Baseline
	"Have you ever seen a general practitioner (GP) for nerves, anxiety, tension or depression?"	F2090	Baseline
**Treatment seeking (assessed in 2022; exploratory longitudinal analyses)**	"Did you ever tell a professional about these problems (medical doctor, psychologist, social worker, counsellor, nurse, clergy, or other helping professional)?" (Depression symptoms)	F29037	MWQ
**Treatment receipt (assessed in 2016; planned and primary analyses)**	"Did you ever try the following for these problems (depression)? (tick all that apply)" (Medication prescribed to you [for at least two weeks])	F20546	MHQ
	"Did you ever use the following for the worry or the problems it caused (anxiety)? (tick all that apply)" (Medication prescribed to you [for at least two weeks])	F20549	MHQ
	"Did you ever try talking therapies for these problems (depression), or other structured activities you regard as therapeutic? Include only those you attended more than once."	F20547	MHQ

	"Did you ever try talking therapies for these problems (anxiety), or other structured activities you regard as therapeutic? Include only those you attended more than once."	F20550	MHQ
**Treatment receipt (assessed in 2022; exploratory longitudinal analyses)**	"Did you ever try the following for these problems? (tick all that apply)" (Medication prescribed to you [for at least two weeks])	F29038	MWQ
	"Which of the following therapies or therapeutic activities have you tried to help you feel better? Include only those you have attended more than once?"	F29047	MWQ
**Other treatment-related behaviours (planned analyses)**	"Have you sought or received professional help for mental distress?"	F20499	MHQ
	"Have you ever been offered/sought treatment for anxiety?"	F21062	DHQ
	"Have you ever been offered/sought treatment for depression?"	F21063	DHQ
**Help-related variables**			
**Informal therapeutic strategies**	"Did you ever try talking therapies for these problems (depression), or other structured activities you regard as therapeutic? Include only those you attended more than once." (Other therapeutic activities such as mindfulness, yoga or art classes)	F20547	MHQ
	"Did you ever try talking therapies for these problems (anxiety), or other structured activities you regard as therapeutic? Include only those you attended more than once." (Other therapeutic activities such as mindfulness, yoga or art classes)	F20550	MHQ
**OTC medication**	"Did you ever try the following for these problems (depression)? (tick all that apply)" (Unprescribed medication [more than once])	F20546	MHQ
	"Did you ever try the following for these problems (anxiety)? (tick all that apply)" (Unprescribed medication [more than once])	F20549	MHQ
**Self-medication with alcohol/drugs**	"Did you ever try the following for these problems (depression)? (tick all that apply)" (Drugs or alcohol [more than once])	F20546	MHQ
	"Did you ever try the following for these problems (anxiety)? (tick all that apply)" (Drugs or alcohol [more than once])	F20549	MHQ
**Helpline**	"Following any time when you took an overdose or deliberately tried to harm yourself did you (tick all that apply):" (use a helpline/voluntary organisation)	F20554	MHQ

**Confiding**	"Following any time when you took an overdose or deliberately tried to harm yourself did you (tick all that apply):" (Receive help from friends/family/neighbours)	F20554	MHQ
**Sociodemographic variables**			
**Sex**	Female/Male	F31	Baseline
**Age**	Age at recruitment	F21022	Baseline
**Ethnicity**	Ethnic background: Responses were recoded into “White British” and “ethnic minority”	F21000	Baseline
**Educational attainment**	"Which of the following qualifications do you have? (You can select more than one)"	F6138	Baseline
**Employment**	Current employment status: "Which of the following describes your current situation? (You can select more than one answer)"	F6142	Baseline
**Household income**	Average total household income before tax: "What is the average total income before tax received by your HOUSEHOLD?"	F738	Baseline
**Neighbourhood deprivation**	Townsend deprivation index calculated at recruitment	F22189	Baseline
**Living arrangement**	"Including yourself, how many people are living together in your household? (Include those who usually live in the house such as students living away from home during term, partners in the armed forces or professions such as pilots)"; "How are the other people who live with you related to you? (You can select more than one answer)"	F709, F6141	Baseline
**Location**	Home area population density	F20118	Baseline
**Emotional support**	"How often are you able to confide in someone close to you?"	F2110	Baseline
**Health-related variables**			
**Perceived overall health**	"In general how would you rate your overall health?"	F2178	Baseline
**Mental health diagnosis**	"Have you been diagnosed with one or more of the following mental health problems by a professional, even if you don't have it currently? (tick all that apply):"	F20544	MHQ
**Psychological distress**	"In your life, have you suffered from a period of mental distress that prevented you from doing your usual activities?"	F20500	MHQ

**Addiction**	"Have you been addicted to or dependent on one or more things, including substances (not cigarettes/coffee) or behaviours (such as gambling)?"	F20401	MHQ
**Number of self-harm methods used**	"Following any time when you took an overdose or deliberately tried to harm yourself did you (tick all that apply):"	F20554	MHQ
**Symptom severity score (covariate)**	"Over the last 2 weeks, how often have you been bothered by any of the following problems? [anxiety symptoms] Becoming easily annoyed or irritable"<br>"Over the last 2 weeks, how often have you been bothered by any of the following problems? [anxiety symptoms] Feeling nervous, anxious or on edge"<br>"Over the last 2 weeks, how often have you been bothered by any of the following problems? [depressive symptoms] Trouble concentrating on things, such as reading the newspaper or watching television"<br>"Over the last 2 weeks, how often have you been bothered by any of the following problems? [anxiety symptoms] Not being able to stop or control worrying"<br>"Over the last 2 weeks, how often have you been bothered by any of the following problems? [depressive symptoms] Feeling down, depressed, or hopeless"<br>"Over the last 2 weeks, how often have you been bothered by any of the following problems? [depressive symptoms] Poor appetite or overeating"<br>"Over the last 2 weeks, how often have you been bothered by any of the following problems? [anxiety symptoms] Feeling afraid as if something awful might happen"<br>"Over the last 2 weeks, how often have you been bothered by any of the following problems? [anxiety symptoms] Trouble relaxing"<br>"Over the last 2 weeks, how often have you been bothered by any of the following problems? [anxiety symptoms] Being so restless that it is hard to sit still"<br>"Over the last 2 weeks, how often have you been bothered by any of the following problems? [depressive symptoms] Trouble falling or staying asleep, or sleeping too much"<br>"Over the last 2 weeks, how often have you been bothered by any of the following problems? [depressive symptoms] Moving or speaking so slowly that other people could	F20505-F20506, F20508-F20512, F20515-F20520	MHQ

	have noticed? Or the opposite - being so fidgety or restless that you have been moving around a lot more than usual"
	"Over the last 2 weeks, how often have you been bothered by any of the following problems? [depressive symptoms] Feeling tired or having little energy"
	"Over the last 2 weeks, how often have you been bothered by any of the following problems? [anxiety symptoms] Worrying too much about different things"

Table S2. Sample characteristics by subgroup and summarised results for group comparisons.

Variable	Sample Characteristics: n (%)					Group Comparisons			
	Total Sample (n=15966)	SI Only (n=5809)	SI with SA (n=822)	Death by Suicide (n=307)	Self-Harm (n=10442)	Overall Group Effect	SI Only vs SI with SA	SI Only vs Death by Suicide	SI with SA vs Death by Suicide
**Sociodemographic**									
**Sex**						*P*<.001*	*P*<.001*	*P*<.001*	*P*<.001*
**Male**	6369 (39.9%)	2657 (45.7%)	314 (38.2%)	211 (68.7%)	3722 (35.6%)				
**Female**	9597 (60.1%)	3152 (54.3%)	508 (61.8%)	96 (31.3%)	6720 (64.4%)				
**Age (years)**						*P*<.001*	*P*<.001*	*P*=1.000	*P*<.001*
**Mean (SD)**	53.14 (7.85)	53.74 (7.89)	51.64 (7.29)	53.84 (8.26)	52.65 (7.77)				
**Ethnicity**						*P*=.224	*P*=.739	*P*=.092	*P*=.091
**White British**	13596 (85.5%)	4909 (84.9%)	687 (84.4%)	271 (84.4%)	7729 (85.8%)				
**Ethnic Minority**	2314 (14.5%)	872 (15.1%)	127 (15.6%)	35 (11.4%)	1281 (14.2%)				
**Missing Data**	55	28	8	0	29				
**Annual Household Income**						*P*<.001*	*P*<.001*	*P*<.001*	*P*=.772
**<£18,000**	3702 (26.0%)	1043 (19.8%)	244 (32.4%)	85 (31.1%)	2330 (29.3%)				
**£18,000 – £31,000**	3550 (24.9%)	1362 (25.8%)	180 (23.9%)	70 (25.6%)	1938 (24.3%)				
**£31,000 – £52,000**	3613 (25.3%)	1426 (27.0%)	178 (23.7%)	70 (25.6%)	1939 (24.4%)				
**>£52,000**	3398 (23.8%)	1444 (27.4%)	150 (19.9%)	48 (17.6%)	1756 (22.1%)				

**Missing Data**	1703	534	70	33	1192				
**Townsend Deprivation Index**						*P*<.001*	*P*<.001*	*P*=.002	*P*=.160
**Mean (SD)**	-.08 (3.36)	-1.02 (3.13)	.17 (3.49)	-.33 (3.41)	-.24 (3.44)				
**Educational Attainment**						*P*=.015	*P*=.052	*P*=.053	*P*=.296
**University**	6431 (45.8%)	2524 (47.4%)	341 (45.3%)	104 (40.3%)	3462 (45.0%)				
**Vocational**	4052 (28.9%)	1505 (28.2%)	194 (25.8%)	76 (29.5%)	2277 (29.6%)				
**Further**	1060 (7.6%)	411 (7.7%)	71 (9.4%)	20 (7.8%)	558 (7.3%)				
**Secondary**	2486 (17.7%)	889 (16.7%)	147 (19.5%)	58 (22.5%)	1392 (18.1%)				
**Missing Data**	1937	480	69	48	1451				
**Employment**						*P*<.001*	*P*<.001*	*P*=.004	*P*<.001*
**Employed**	8697 (55.0%)	3334 (58.0%)	415 (50.8%)	173 (57.1%)	4775 (53.4%)				
**Retired**	4360 (27.6%)	1754 (30.5%)	167 (20.4%)	77 (25.4%)	2362 (26.4%)				
**Unemployed**	2756 (17.4%)	665 (11.5%)	235 (28.8%)	53 (17.5%)	1803 (20.2%)				
**Missing Data**	153	56	5	3	98				
**Living Arrangement**						*P*<.001*	*P*<.001*	*P*<.001*	*P*=.077
**Alone**	4037 (25.4%)	1242 (21.4%)	259 (31.6%)	97 (31.9%)	2439 (27.2%)				
**Partner**	9750 (61.4%)	3847 (66.3%)	420 (51.2%)	171 (56.2%)	5312 (59.3%)				

**Other**	2102 (13.2%)	712 (12.3%)	141 (17.2%)	36 (11.8%)	121 (13.5%)				
**Missing Data**	78	8	2	2	68				
**Location**						*P*=.017	*P*=.006	*P*=.204	*P*=.788
**Urban**	13849 (87.9%)	4991 (87.2%)	732 (90.6%)	273 (89.8%)	7853 (88.1%)				
**Rural**	1900 (12.1%)	734 (12.8%)	76 (9.4%)	31 (10.2%)	1059 (11.9%)				
**Missing Data**	217	84	14	2	137				
**Confiding**						*P*<.001*	*P*=.002	*P*=.005	*P*<.001*
**No**	3464 (22.2%)	1455 (25.5%)	248 (30.7%)	54 (18.2%)	1707 (19.3%)				
**Yes**	12165 (77.8%)	4240 (74.5%)	560 (69.3%)	243 (81.8%)	7122 (80.7%)				
**Missing Data**	337	114	14	9	223				
**Health-Related**									
**Perceived Overall Health**						*P*<.001*	*P*<.001*	*P*=.505	*P*<.001*
**Excellent**	1645 (10.4%)	598 (10.3%)	55 (6.7%)	35 (11.7%)	957 (10.7%)				
**Good**	7724 (48.7%)	2894 (50.0%)	304 (37.2%)	158 (52.7%)	4368 (48.8%)				
**Fair**	4522 (28.5%)	1675 (28.9%)	263 (32.2%)	76 (25.3%)	2508 (28.0%)				
**Poor**	1978 (12.5%)	625 (10.8%)	195 (23.9%)	31 (10.3%)	1127 (12.6%)				
**Missing Data**	97	17	5	6	77				
**Mental Health Diagnosis**						*P*<.001*	*P*<.001*	N/A	N/A
**No**	4006 (32.7%%)	2175 (37.7%)	88 (10.9%)	8 (28.6%)	1735 (30.8%)				

**Yes**	8247 (67.3%)	3598 (62.3%)	723 (89.1%)	20 (71.4%)	3906 (69.2%)				
**Missing Data**	3713	36	11	278	3463				
**Psychological Distress**						*P*<.001*	*P*<.001*	N/A	N/A
**No**	3350 (27.6%)	1841 (32.6%)	48 (5.9%)	7 (24.1%)	1454 (25.8%)				
**Yes**	8782 (72.4%)	3800 (67.4%)	769 (94.1%)	22 (75.9%)	4191 (74.2%)				
**Missing Data**	3834	168	5	277	3451				
**Addiction**						*P*<.001*	*P*<.001*	N/A	N/A
**No**	9879 (82.0%)	4747 (83.8%)	552 (70.2%)	24 (82.8%)	4556 (81.9%)				
**Yes**	2164 (18.0%)	917 (16.2%)	234 (29.8%)	5 (17.2%)	1008 (18.1%)				
**Missing Data**	3923	145	36	277	3580				
**Number of Self-Harm Methods Used (0-6)**						*P*<.001*	*P*<.001*	N/A	N/A
**Mean (SD)**	2.38 (0.70)	2.37 (0.67)	2.78 (0.94)	2.91 (1.14)	2.32 (0.63)				
**Help-Related**									
**Informal Therapeutic Strategies**						*P*<.001*	*P*<.001*	N/A	N/A
**No**	5186 (69.6%)	2233 (70.4%)	329 (61.8%)	0 (0.0%)	2624 (70.0%)				
**Yes**	2267 (30.4%)	938 (29.6%)	203 (38.2%)	0 (0.0%)	1127 (30.0%)				
**Missing Data**	8513	2638	290	307	5799				
**OTC Medication**						*P*<.001*	*P*<.001*	N/A	N/A
**No**	6740 (90.8%)	2854 (90.3%)	468 (88.1%)	0 (0.0%)	3418 (91.5%)				

**Yes**	689 (9.3%)	308 (9.7%)	63 (11.9%)	0 (0.0%)	318 (8.5%)				
**Missing Data**	8537	2647	291	307	5814				
**Self-Medication with Alcohol/Drugs**						*P*<.001*	*P*<.001*	N/A	N/A
**No**	7928 (70.8%)	3881 (73.4%)	439 (58.1%)	15 (60.0%)	3593 (69.9%)				
**Yes**	3282 (34.5%)	1410 (26.6%)	316 (41.9%)	10 (40.0%)	1546 (30.1%)				
**Missing Data**	4756	518	67	281	3969				
**Helpline (e.g., helplines)**						*P*<.001*	*P*<.001*	N/A	N/A
**No**	6461 (90.3%)	448 (92.9%)	681 (82.8%)	30 (93.8%)	5302 (91.2%)				
**Yes**	691 (9.7%)	34 (7.05%)	141 (17.2%)	2 (6.2%)	514 (8.8%)				
**Missing Data**	8814	5327	141	275	3308				
**Interpersonal Support**						*P*<.001*	*P*<.001*	N/A	N/A
**No**	5130 (71.7%)	399 (82.8%)	507 (61.7%)	24 (75.0%)	4200 (72.2%)				
**Yes**	2022 (29.3%)	83 (17.2%)	315 (38.3%)	8 (25.0%)	1616 (27.8%)				
**Missing Data**	8814	5327	315	275	3308				
**Treatment-Related Behaviours**									
**Treatment-Seeking**						*P*<.001*	*P*<.001*	N/A	N/A
**No**	2146 (19.1%)	1307 (24.7%)	58 (7.2%)	3 (11.5%)	895 (13.9%)				
**Yes**	9103 (80.9%)	3976 (75.3%)	753 (92.8%)	23 (88.5%)	5565 (86.1%)				
**Missing Data**	4717	526	11	281	3899				
**Treatment Receipt**						*P*<.001*	*P*<.001*	N/A	N/A

**No**	2738 (24.2%)	1588 (30.0%)	107 (13.0%)	5 (19.2%)	1179 (18.2%)				
**Yes**	8554 (75.8%)	3714 (70.0%)	715 (87.0%)	21 (80.8%)	5298 (81.8%)				
**Missing Data**	4674	507	0	281	3886				
**Other Treatment-Related Behaviours**						*P*<.001*	*P*<.001*	N/A	N/A
**No**	3064 (24.8%)	1790 (30.9%)	58 (7.1%)	7 (23.3%)	1333 (18.9%)				
**Yes**	9272 (75.2%)	4000 (69.1%)	763 (92.9%)	23 (76.7%)	5718 (81.1%)				
**Missing Data**	3630	19	1	277	3333				

Note: The sum of sample sizes across the four subgroups (SI Only, SI with SA, Death by Suicide and Self-harm) exceeds the total sample size (n=15966). This is because there are overlaps between the Self-harm subgroups and the other subgroups. While SI Only, SI with SA, and Death by Suicide are mutually exclusive categories, some individuals in the Self-harm subgroup may also belong to the SI Only, SI with SA, or Death by Suicide subgroup. Thus, comparisons were only made between SI Only, SI with SA, and Death by Suicide subgroups. The Death by Suicide subgroup had a high proportion (90.2%) who did not complete the MHQ, resulting in insufficient data on treatment-related behaviours and other variables assessed through the MHQ. As such, variables assessed through the MHQ were not compared between Death by Suicide and the other subgroups.

Table S3. Contingency table displaying the absence of a mental health diagnosis (vs. presence) in relation to treatment seeking as well as treatment receipt among all participants.

Mental Health Diagnosis	Treatment Seeking		Treatment Receipt	
	Non-Seeking	Seeking	Non-Receipt	Receipt
Absence	1673	1243	1978	958
Presence	321	6585	539	6378

Table S4. Odds ratios and 95% confidence intervals from planned multivariable logistic regression models assessing factors associated with reductions in treatment-related behaviours in the planned analyses.

	Total Sample (n=15966)			SI Only (n=5809)			Self-harm (n=10442)		
**Variable**	**Treatment Seeking**	**Treatment Receipt**	**Other Treatment-Related Behaviours**	**Treatment Seeking**	**Treatment Receipt**	**Other Treatment-Related Behaviours**	**Treatment Seeking**	**Treatment Receipt**	**Other Treatment-Related Behaviours**
**Sex (Ref = Female)**									
**Male**	**.65 [.53, .81]**	**.58 [.47, .70]**	**.71 [.57, .88]**	**.69 [.56, .84]**	**.62 [.51, .76]**	**.68 [.56, .84]**	**.53 [.42, .68]**	**.54 [.44, .68]**	.71 [.55, .91]
**Age (years)**	1.01 [.99, 1.02]	1.01 [1.00, 1.03]	1.00 [.98, 1.02]	1.00 [.98, 1.02]	1.00 [.98, 1.01]	.99 [.97, 1.00]	1.00 [.99, 1.02]	1.01 [.99, 1.03]	1.00 [.98, 1.02]
**Ethnicity (Ref = White British)**									
**Ethnic Minority**	.81 [.62, 1.06]	.88 [.69, 1.13]	.92 [.70, 1.21]	1.20 [.92, 1.57]	1.21 [.93, 1.58]	1.29 [.97, 1.70]	.82 [.61, 1.10]	.85 [.65, 1.12]	.96 [.71, 1.31]
**Annual Household Income (Ref = <£18 000)**									
**£18 000–£31 000**	1.53 [1.07, 2.18]	1.43 [1.04, 1.97]	1.27 [.89, 1.81]	1.19 [.85, 1.65]	1.16 [.85, 1.59]	1.14 [.82, 1.60]	1.19 [.80, 1.77]	1.00 [.71, 1.42]	1.26 [.84, 1.89]
**£31 000–£52 000**	1.12 [.78, 1.59]	1.23 [.88, 1.70]	1.27 [.88, 1.82]	1.04 [.74, 1.46]	1.12 [.80, 1.55]	1.16 [.82, 1.65]	1.00 [.67, 1.50]	.89 [.62. 1.27]	1.12 [.74, 1.70]
**>£52 000**	1.46 [1.00, 2.14]	1.48 [1.05, 2.10]	1.68 [1.14, 2.48]	1.22 [.85, 1.76]	1.25 [.88, 1.77]	1.46 [1.00, 2.12]	1.51 [.98, 2.32]	1.19 [.80, 1.74]	1.58 [1.01, 2.47]
**Townsend Deprivation Index**	1.02 [.98, 1.05]	1.01 [.98, 1.04]	1.03 [1.00, 1.07]	1.00 [.97, 1.04]	1.03 [1.00, 1.07]	1.03 [1.00, 1.07]	1.01 [.98, 2.32]	1.00 [.97, 1.04]	1.01 [.97, 1.05]
**Educational Attainment (Ref = University)**									
**Vocational**	.86 [.67, 1.10]	.75 [.60, .94]	.75 [.58, .96]	.97 [.76, 1.23]	.75 [.59, .94]	.82 [.64, 1.05]	.92 [.71, 1.22]	.87 [.68, 1.12]	.73 [.55, .97]
**Further**	.98 [.67, 1.45]	.90 [.63, 1.27]	.90 [.61, 1.33]	1.14 [.79, 1.66]	.92 [.64, 1.31]	.94 [.64, 1.37]	1.10 [.70, 1.75]	.96 [.65, 1.45]	1.03 [.65, 1.67]
**Secondary**	.75 [.54, 1.03]	.88 [.65, 1.18]	.75 [.54, 1.04]	.73 [.55, .98]	.78 [.59, 1.03]	.69 [.51, .93]	1.03 [.72, 1.48]	.79 [.57, 1.08]	.86 [.60, 1.25]
**Employment (Ref = Employed)**									
**Retired**	1.11	1.08	1.01	.93	1.03	1.07	1.15	1.07	.98

	[.83, 1.48]	[.83, 1.41]	[.76, 1.35]	[.71, 1.23]	[.79, 1.35]	[.81, 1.42]	[.84, 1.60]	[.80, 1.45]	[.70, 1.37]
**Unemployed**	1.12	1.14	.89	.84	1.22	1.04	1.05	.96	.94
	[.76, 1.67]	[.81, 1.63]	[.61, 1.32]	[.58, 1.21]	[.85, 1.75]	[.71, 1.53]	[.68, 1.65]	[.66, 1.41]	[.61, 1.50]
**Living Arrangement (Ref = Alone)**									
**Partner**	.81	**.66**	.69	.74	**.61**	.69	1.01	.89	.71
	[.83, 1.48]	**[.51, .85]**	[.52, .92]	[.56, .97]	**[.46, .79]**	[.53, .92]	[.74, 1.39]	[.67, 1.19]	[.51, .99]
**Other**	.80	.76	.89	.77	.71	.80	1.14	.95	.78
	[.55, 1.15]	[.54, 1.06]	[.61, 1.30]	[.53, 1.12]	[.50, 1.02]	[.55, 1.18]	[.76, 1.73]	[.66, 1.37]	[.51, 1.19]
**Location (Ref = Urban)**									
**Rural**	.92	1.03	.93	1.03	.90 [.69,	.87	.81	1.01	1.16
	[.68, 1.25]	[.78, 1.37]	[.69, 1.27]	[.78, 1.38]	1.19]	[.65, 1.17]	[.58, 1.13]	[.75, 1.38]	[.82, 1.65]
**Confiding (Ref = Presence)**									
**Absence**	.70	.74	.75	.76	.78	.75	.77	.68	.88
	[.55, .89]	[.59, .93]	[.59, .96]	[.60, .94]	[.63, .97]	[.59, .94]	[.58, 1.03]	[.52, .89]	[.65, 1.19]
**Perceived Overall Health (Ref = Good)**									
**Excellent**	.93	1.07	1.00	1.13	.95	1.09	.76	.95	.93
	[.70, 1.23]	[.82, 1.40]	[.74, 1.35]	[.84, 1.63]	[.71, 1.27]	[.80, 1.48]	[.55, 1.01]	[.72, 1.28]	[.68, 1.28]
**Fair**	1.21	1.41	1.25	1.23	1.28	1.09	1.08	1.18	.99
	[.94, 1.57]	[1.12, 1.78]	[.96, 1.62]	[.97, 1.57]	[1.02, 1.60]	[.85, 1.39]	[.81, 1.45]	[.91, 1.53]	[.73, 1.34]
**Poor**	1.17	1.81	1.14	1.10	1.38	.98	1.02	1.25	.74
	[.76, 1.85]	[1.21, 2.77]	[.74, 1.80]	[.78, 1.38]	[.96, 2.00]	[.67, 1.46]	[.61, 1.73]	[.80, 2.00]	[.44, 1.25]
**Mental Health Diagnosis (Ref = Presence)**									
**Absence**	**.04**	**.05**	**.04**	**.06**	**.06**	**.05**	**.04**	**.05**	**.04**
	**[.03, .05]**	**[.04, .06]**	**[.03, .05]**	**[.04, .07]**	**[.05, .07]**	**[.04, .06]**	**[.03, .06]**	**[.04, .06]**	**[.03, .05]**
**Psychological Distress (Ref = Presence)**									
**Absence**	**.41**	**.48**	**.31**	**.36**	**.44**	**.25**	**.45**	**.48**	**.28**
	**[.34, .50]**	**[.39, .60]**	**[.25, .39]**	**[.29, .44]**	**[.36, .54]**	**[.20, .31]**	**[.36, .57]**	**[.38, .60]**	**[.22, .35]**
**Addiction (Ref = Presence)**									
**Absence**	1.05	.95	1.09	1.05	1.03	1.11	.78	.76	.69
	[.79, 1.41]	[.73, 1.24]	[.81, 1.45]	[.78, 1.41]	[.78, 1.37]	[.82, 1.49]	[.93, 1.77]	[.44, 1.27]	[.49, .95]
**Informal Therapeutic Strategies (e.g., yoga; Ref = Use)**									
**Non-use**	**.65**	**.66**	**.55**	**.58**	.68	**.58**	.66	.83	**.57**
	**[.52, .83]**	**[.53, .83]**	**[.42, .70]**	**[.45, .75]**	[.53, .87]	**[.44, .76]**	[.49, .89]	[.65, 1.08]	**[.42, .68]**
**OTC Medication (Ref = Use)**									
**Non-use**	.99	.92	1.15	.87	.76	.88	1.59	.96	1.30
	[.67, 1.45]	[.63, 1.28]	[.78, 1.69]	[.61, 1.22]	[.67, 1.05]	[.63, 1.25]	[1.11, 2.22]	[.69, 1.32]	[.89, 1.89]
**Self-Medication with Alcohol/Drugs (Ref = Use)**									

**Non-use**	.79 [.62, 1.00]	.84 [.68, 1.04]	.72 [.57, .93]	.99 [.78, 1.25]	.84 [.67, 1.05]	.84 [.66, 1.08]	1.08 [.82, 1.39]	1.15 [.90, 1.45]	.98 [.75, 1.28]
**Helpline (e.g., helplines; Ref = Use)**									
**Non-use**	N/A	N/A	N/A	N/A	N/A	N/A	**.24 [.12, .45]**	**.29 [.16, .48]**	**.20 [.09, .39]**
**Interpersonal Support (e.g., family; Ref = Use)**									
**Non-use**	N/A	N/A	N/A	N/A	N/A	N/A	.87 [.66, 1.14]	.90 [.70, 1.15]	1.01 [.76, 1.33]
**Number of Self-Harm Methods Used (0-6)**	N/A	N/A	N/A	N/A	N/A	N/A	1.17 [.95, 1.44]	1.27 [1.06, 1.53]	1.14 [.92, 1.42]

Note: Self-harm-related variables (i.e., helpline for self-harm, interpersonal support for self-harm, and number of self-harm methods used) were only available for participants who reported self-harm behaviours, and thus were assessed as potential predictors only in the Self-harm subgroup. Bold values denotes significance based on a Bonferroni-corrected *P* threshold of .002.

Supplementary Figures

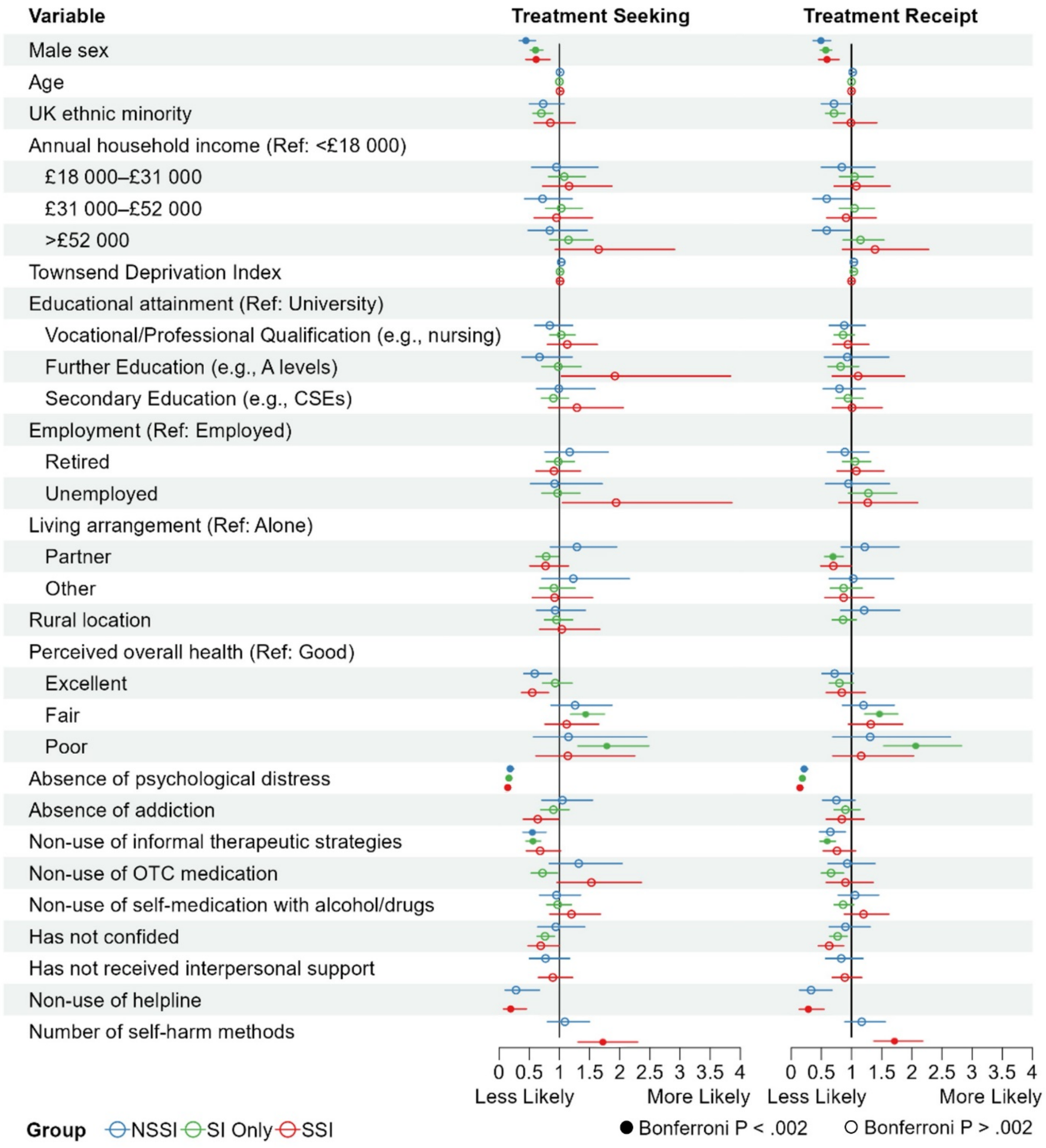

Figure S1. Forest plots showing the odds ratios (with 95% confidence intervals) for each factor in the multivariable models, examining their associations with treatment seeking and treatment receipt respectively, across the NSSI, SI Only and SSI subgroups.

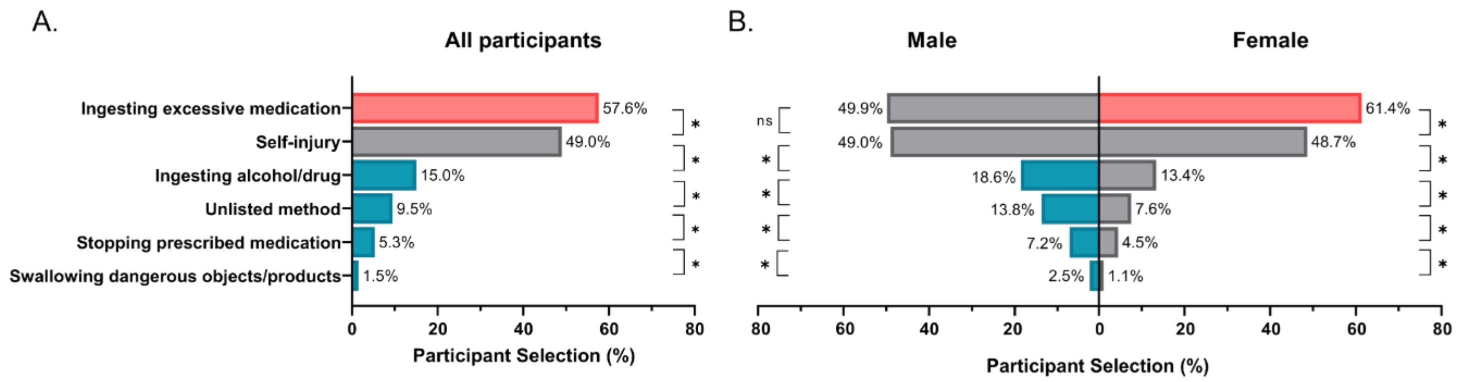

Figure S2. Percentage of participants using each self-harm method: All participants (A), and by Sex (B). Red bars represent methods used significantly more frequently by females than males; blue bars indicate methods used significantly more frequently by males than females. The asterisks (*) denote statistically significant differences in the pairwise comparisons between self-harm methods, where 'ns' indicates nonsignificant results.

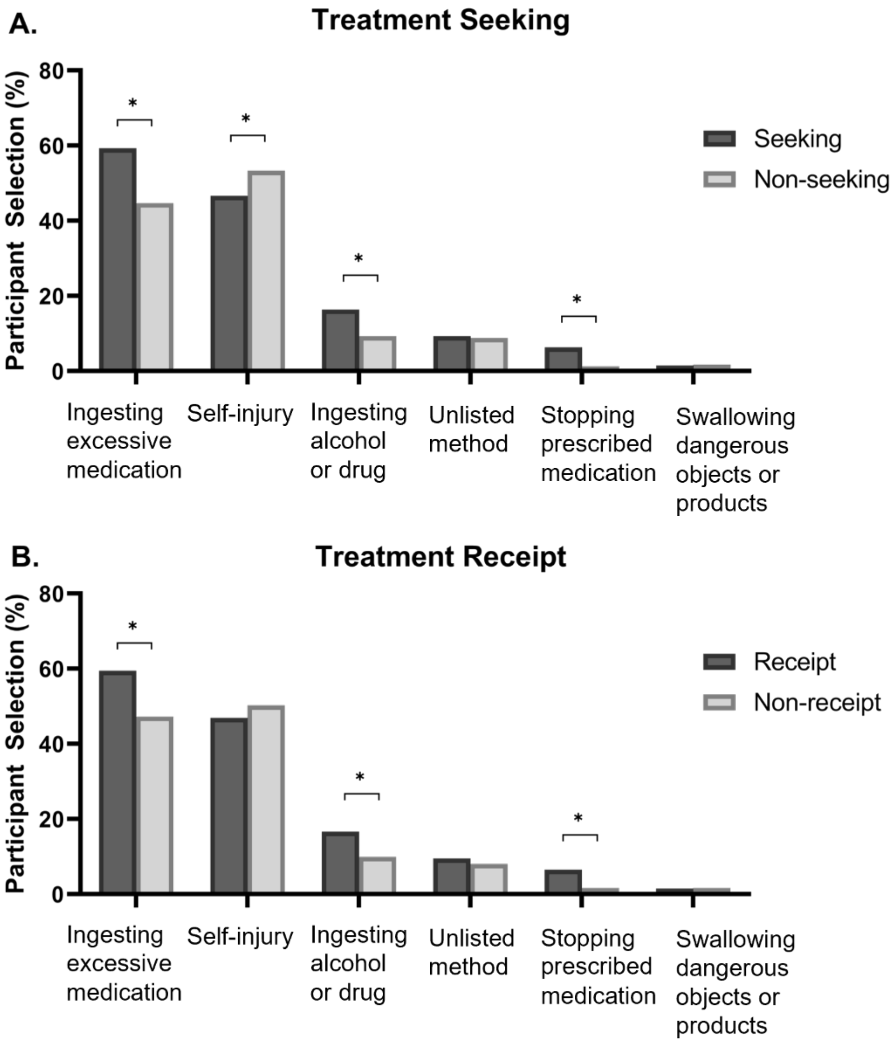

Figure S3. Use of self-harm methods among treatment-seekers/non-seekers (A) and treatment receivers/non-receivers (B). The asterisks (*) denote statistically significant differences in the pairwise comparisons between groups.
